# Spatiotemporal dynamics of carbon imbalance in agricultural cultivation and its driving factors: a study based on Hunan Province, China

**DOI:** 10.1186/s13021-026-00413-9

**Published:** 2026-02-01

**Authors:** Chenxi Dou, Xianzhao Liu, Jiaxi Liu, Yue Xing, Hai Xiao, Sixiang Quan

**Affiliations:** 1 School of Earth Science and Space Information Engineering, Hunan University of Science and Technology, Xiangtan, 411201 China; 2 School of Economics, Guangxi Minzu University, Nanning, 530006 China; 3 Hunan Engineering Research Center of Natural Ecosystems Carbon Sink Monitoring, Changsha, 410000 China

**Keywords:** Agricultural cultivation, Carbon imbalance, Spatiotemporal pattern, Driving factor, Sustainable development

## Abstract

**Background:**

Against the backdrop of global warming, the imbalance in agricultural carbon budgets poses a dual threat to ecological security and food security. As a major grain-producing region in China, Hunan Province is confronted with substantial CH_4_ emissions derived from rice cultivation, a problem further exacerbated by industrialization, urbanization, and shifts in farming practices. Consequently, investigating the carbon imbalance in Hunan’s agricultural cultivation is of great significance for advancing the sustainable development of agriculture in the province. This study constructs and quantifies the agricultural carbon imbalance index (CII), and employs exploratory spatiotemporal data analysis, the PLS-VIP method, and the GTWR model to analyze the spatiotemporal evolution and driving factors of agricultural cultivation carbon imbalance of Hunan Province in China from 2001 to 2022.

**Results:**

(1) The CII for agricultural cultivation in Hunan Province decreased from 0.41 in 2001 to 0.26 in 2022. Its spatiotemporal pattern shifted from “high in the north and low in the south” to “high in the west and low in the east,” with the gravity center of CII moving southwestward. (2) Over the study period, the spatial correlation characteristics of CII underwent three stages: significant positive correlation, random distribution, and weak positive correlation. LISA time path and spatiotemporal transition analyses showed that the spatiotemporal clustering pattern of CII remained relatively stable from 2001 to 2011; however, its stability weakened slightly from 2012 to 2022. (3) Key factors influencing the agricultural cultivation CII in Hunan Province include GPA, GST, IARDF, PAOV, FUI, and PUI. These factors exhibit significant spatiotemporal heterogeneity in their effects. For example, the FUI and PUI had significant impacts on CII in the Xiangbei region, whereas their influence was relatively weaker in the Xiangxi region.

**Conclusions:**

To alleviate the persistent carbon imbalance in Hunan’s agricultural cultivation systems, differentiated carbon sequestration and emission reduction strategies should be formulated by integrating the significance hierarchy of CII drivers and their spatial heterogeneity patterns. Special emphasis ought to be placed on tackling the re-emergence of carbon imbalance in specific municipal regions, which stems from urban expansion encroaching on farmland and the persistence of traditional cultivation practices. This targeted optimization will effectively facilitate the sustainable and low-carbon development of Hunan’s agricultural sector.

## Introduction

Against the backdrop of global climate warming, the ecological and environmental risks arising from carbon budget imbalances have become one of the core challenges constraining the sustainable development of humankind. The Intergovernmental Panel on Climate Change (IPCC) explicitly stated in its Special Report on Global Warming of 1.5 °C that if the current rate of greenhouse gas emissions continues unchanged, global temperatures are projected to rise by 1.5 °C between 2030 and 2052, further exacerbating global warming. In this context, effectively addressing carbon budget imbalances has become a critical challenge for governments around the world in their efforts to mitigate global warming. As the world’s largest carbon emitter, China’s carbon emissions surpassed 12.6 billion tons in 2024, accounting for approximately 35% of global total carbon emissions [1]. Of this total, agricultural carbon emissions reached 828 million tons, representing about 6.7% of the national total [2]. As a basic industry of the national economy, agriculture is both an important source of carbon emissions and an important carbon sequestration (e.g., China’s total agricultural sequestration reached 106 million tons in 2021), which is a key link for China to realize the goal of “double carbon”. In the agricultural sector, carbon emissions from agricultural cultivation account for approximately 67% of total agricultural carbon emissions [3], highlighting substantial potential for carbon sequestration and emission reduction in China’s agricultural cultivation. Meanwhile, the food demand of China’s 1.4 billion people relies heavily on outputs from agricultural cultivation, which makes agricultural cultivation in the realization of carbon sequestration and emission reduction at the same time also shoulder the important mission of ensuring national food security [4]. In particular, with the improvement of living standards and consumption upgrading leading to a steep increase in food demand, coupled with the rapid industrialization and urbanization rigidly compressing arable land area, exacerbating the fragmentation of arable land, farmers are forced to invest in a large number of agricultural materials (chemical fertilizers, pesticides, agricultural films, etc.) in order to maintain the yield, which leads to significant changes in cultivation patterns and production scales [5–7]. Estimates from Dong et al. indicate that CH₄ and N₂O emissions from agricultural cultivation activities in China will increase by more than 35% by 2030 [8]. This suggests that the risk of carbon imbalance in agricultural cultivation may be further exacerbated. The long-term imbalance between carbon emissions and carbon sequestration in agricultural cultivation not only depletes soil organic carbon pools and degrades arable land quality but also, more importantly, undermines the sustainable development of agro-ecosystems, posing a dual threat to food security and ecological security. Moreover, current assessments of the agricultural carbon budget primarily focus on various types of energy consumption during agricultural production, which limits their ability to accurately capture the dynamics of carbon imbalance between agricultural inputs and the crop-soil system. In particular, there is a lack of quantitative research on the regional differences in the contribution magnitudes of carbon emissions and carbon sequestration to carbon imbalance. Meanwhile, the agricultural carbon footprint may also exhibit significant spatiotemporal heterogeneity due to variations in natural conditions, levels of economic development, and cultivation structures across different regions [9].To address the above issues, this paper takes Hunan Province, a typical rice-producing area in China, as an example and constructs a carbon imbalance index (CII) for agricultural cultivation based on the relative differences and temporal dynamics of the carbon budget in the study area. By examining the spatiotemporal pattern evolution and driving mechanisms of carbon imbalance in agricultural cultivation, this study aims to provide theoretical support and empirical evidence for the precise assessment of agricultural carbon imbalance and the promotion of agricultural sustainable development.

Whether the agricultural cultivation carbon budget is imbalanced is primarily determined by the relationship between carbon emissions and carbon sequestration in agricultural cultivation. Currently, research on agricultural cultivation carbon imbalances focuses on measurement methods [10, 11], spatiotemporal patterns [12, 13], and driving factors [11, 14]. Regarding measurement methods, early scholars used the Kaya-Zenga index and the MRIO model to analyze the carbon imbalance caused by provincial carbon emissions and trade-induced carbon transfer in China. However, these studies failed to consider the impact of carbon sequestration when characterizing carbon imbalance [15–17]. Subsequently, some scholars integrated carbon sequestration into carbon imbalance assessments, measuring carbon imbalance by means of the difference or ratio of carbon emissions to carbon sequestration [11, 18]. Although this approach is intuitive, it overlooks the dynamic changes in carbon imbalance from year to year. Other scholars have employed the carbon emission economic contribution coefficient (ECC) and carbon ecological support coefficient (ESC) based on ecological functional zoning to assess carbon imbalance from the perspectives of regional carbon productivity and carbon sequestration capacity [19–21]. For example, Lu et al. employed the ESC to construct the static carbon balance index (FSBI) and dynamic carbon balance index (FDBI) to measure and conduct zoning of the carbon balance of grain production in the Yellow River Basin [22]. In addition, Ji et al. introduced risk assessment methods from the field of economics into carbon imbalance measurement and analyzed carbon imbalance issues at the county and grid scales in Henan Province by constructing a carbon imbalance risk index, enhancing the calculation accuracy and risk management capabilities of carbon imbalances [10]. However, existing studies have yet to reach a consensus on the measurement methods for carbon imbalance.

Regarding spatiotemporal patterns, Jing et al. employed spatial visualization tools to analyze the spatiotemporal characteristics of land use carbon budgets in Chinese provinces [23]. They found that China’s arable land carbon budgets exhibit an “east high, west low” spatial distribution pattern, and concluded that economic development levels and geographical environmental factors are the primary drivers of inter-provincial spatial differences in carbon budgets; Wu et al. used the Dagum Gini coefficient and spatial Markov chain methods to analyze the spatial disparities and evolutionary mechanisms of land use carbon budgets in Fujian Province of China, revealing an intensifying phenomenon of carbon budget imbalance alongside strong spatial spillover effects of carbon emissions [24]; Han et al. utilized exploratory spatial data analysis tools to examine spatiotemporal distribution features of agricultural carbon budgets at the county level in Hunan Province of China [25]. Their findings indicated significant positive spatial correlation in agricultural carbon budgets across Hunan Province, with carbon emissions displaying a “central high, peripheral gradual decrease” pattern and carbon sequestration showing a distribution of “high in the east, central, and northern regions and low in the southwest.” Although the above scholars have made many useful explorations into the spatiotemporal traits of carbon budgets, there has been little research that delves deeply into the spatial aggregation characteristics and dynamic evolution of carbon imbalances in agricultural cultivation from the perspective of carbon budgets.

Regarding research on the driving factors of carbon imbalance, Liu et al. and Nie et al. employed the Spatial Durbin Models to analyze the influencing factors of carbon imbalance in Chinese provinces and the Yellow River basin, respectively [11, 26]. These studies highlighted that population size, economic growth, and the proportion of the secondary industry are statistically positively correlated with carbon imbalance, while agricultural mechanization efficiency exerts a significant inhibitory effect on agricultural carbon imbalance. Zhong et al. examined the spatiotemporal distribution of carbon imbalance in counties of Hunan Province in China, identifying economic structure, policy effectiveness, and geographical patterns as key factors influencing carbon imbalance in the province [13]. Similarly, Hua and Jing analyzed the impacts of various socioeconomic factors on carbon balance and imbalance based on the eco-economic coordination index [14]. Their findings showed that urbanization level positively drives carbon imbalance, while total population, regional GDP, secondary industry added value, and coal consumption exert a negative effect. Yang and Su (2025) employed the GTWR model to examine factors influencing carbon balance and imbalance in the Guanzhong Plain urban agglomeration, revealing that energy intensity, GDP, industrial structure, and urbanization level promote carbon imbalance, while climatic factors such as precipitation inhibit it [7]. Additionally, the impacts of these driving factors exhibit significant spatiotemporal heterogeneity. Although the above studies have identified the main factors influencing carbon imbalance, research on the spatiotemporal heterogeneity of driving factors in agricultural cultivation carbon imbalance remains limited. Notably, the factors driving agricultural cultivation carbon imbalance are multifaceted and complex, shaped not only by regional disparities in natural conditions but also compounded by the combined effects of national agricultural policies, socioeconomic conditions, and irrigation and fertilization management practices [10].

From a spatial perspective, existing studies have primarily focused on the watershed, provincial, and county levels [23–25], while research at the municipal level relatively limited. In fact, the municipal scale plays a central role in achieving agricultural sustainable development, and findings from macro- or micro-level studies are insufficient to support the formulation of policies for agricultural sustainable development at the municipal level. Therefore, examining the issue of carbon imbalance in agricultural cultivation activities at the municipal level will assist relevant authorities in formulating targeted and refined agricultural carbon sequestration and emission reduction measures, thereby advancing agricultural sustainable development.

In summary, although extensive research has been conducted on carbon imbalance from the perspectives of measurement methods, spatiotemporal patterns, and driving factors, three main limitations remain. First, most existing studies characterize carbon imbalance using static differences or ratios between carbon emissions and carbon sequestration, which makes it difficult to capture temporal variations in carbon imbalance. Therefore, there is an urgent need to develop a dynamic index that can comprehensively reflect both the relative differences between carbon emissions and carbon sequestration and their rates of change. Second, with respect to spatiotemporal patterns, previous studies have mainly focused on the spatiotemporal distribution of agricultural carbon budgets, while lacking systematic investigation into the spatial clustering, spatiotemporal changes, and dynamic transition processes of carbon imbalance. Finally, regarding driving factors, although existing literature has identified the effects of population, economic, and technological factors on carbon imbalance at the watershed or provincial scale, studies on the spatiotemporal heterogeneity of the driving factors of agricultural planting carbon imbalance at the municipal scale remain relatively scarce. Based on the above analysis, this paper constructs and calculates the agricultural cultivation carbon imbalance index (CII) for each city-level region in Hunan Province of China from 2001 to 2022, and explores its spatiotemporal pattern evolution characteristics and driving factors. Compared with existing research, the main contributions of this paper are as follows: (1) The concept of carbon imbalance was introduced into the field of agricultural cultivation, and the CII was constructed by comprehensively considering the differences between carbon emissions and carbon sequestration as well as their fluctuation rates. This provides a feasible method for the comprehensive quantitative assessment of carbon imbalance in agricultural cultivation.​ (2) Based on the CII, the spatiotemporal evolution patterns and main causes of agricultural cultivation carbon imbalance in Hunan Province were systematically investigated. Furthermore, grounded in the theory of sustainable development, the practical effects of agricultural policy transformation on mitigating carbon imbalance in this region were further analyzed. ​(3) The PLS-VIP and GTWR models were employed to identify and analyze the key driving factors of agricultural cultivation carbon imbalance at the municipal level in Hunan Province, as well as the spatiotemporal heterogeneity of their impacts on carbon imbalance. This provides a theoretical basis for agricultural and related sectors to formulate differentiated carbon sequestration and emission reduction policies.

## Research area overview

**Fig. 1:**
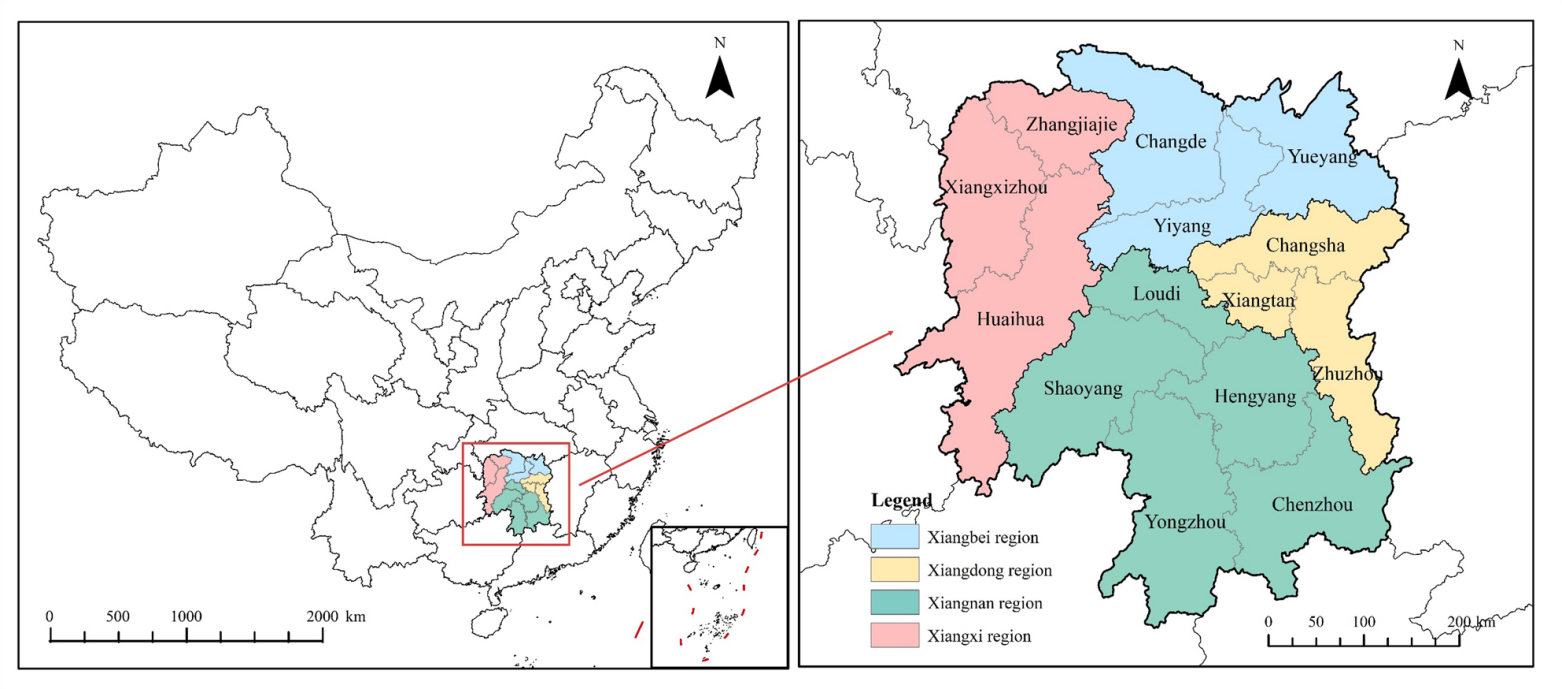
Schematic map of the study area

Hunan Province is located between 24°38′−30 °08′N and 108°47′−114°15′E. It has a subtropical monsoon climate with abundant sunlight, plentiful rainfall, and rainfall coinciding with the growing season, providing extremely favorable climatic conditions for crop growth. As a result, Hunan Province has become one of China’s important grain-producing regions and grain production bases. According to existing research statistics, in 2024, Hunan Province’s grain cultivation area was 4.667 million hectares, with rice cultivation area reaching 3.6 million hectares and grain production exceeding 26.75 billion kilograms [25]. Hunan Province produced 4.4% of the country’s grain with only 2.8% of the country’s arable land, playing a pivotal role in ensuring national food security. However, the large-scale cultivation of rice produces significant CH_4_ emissions, which may exacerbate the risk of carbon imbalance in agricultural cultivation in Hunan Province [27, 28]. According to research, Hunan Province’s agricultural carbon emissions in 2020 were approximately 79.4 million tons CO_2_, while carbon sequestration was only 36.4 million tons CO_2_ [25], with the ratio of the former to the latter being 2.18. The situation of high carbon emissions and low carbon sequestration coexisting is typical of most agriculture in China, so it is scientifically significant to discuss the imbalance between carbon emissions and carbon sequestration in Hunan Province’s agriculture. For the convenience of research, this paper divides Hunan Province into four regions based on the geographical locations of its city administrative units (Fig. 1), namely the Xiangbei region (Changde, Yiyang, and Yueyang), the Xiangdong region (Changsha, Zhuzhou, and Xiangtan), the Xiangnan region (Shaoyang, Loudi, Hengyang, Yongzhou, and Chenzhou), and the Xiangxi region (Zhangjiajie, Xiangxizhou, and Huaihua).

## Research methods and data sources

### Definition of carbon imbalance in agricultural cultivation

Carbon imbalance is an important indicator for measuring the relationship between carbon emissions and carbon sequestration in the research area, but there are currently differences in understanding its meaning within the academic community. Some scholars believe that if the carbon emissions in a study area exceed the carbon sequestration during the same period, it is considered a carbon imbalance; otherwise, it is considered a carbon balance [29]. Another group of scholars adopts a dynamic equilibrium perspective, using the magnitude of changes in carbon emissions and carbon sequestration during the same period to characterize carbon imbalance. Specifically, when the growth rate of carbon emissions in a certain region exceeds the growth rate of carbon sequestration during the study period, it is considered a carbon imbalance; conversely, it is considered a carbon balance [22]. From the perspective of agricultural cultivation systems, when carbon emissions are less than or equal to carbon sequestration, the system exhibits a certain capacity for self-regulation and carbon-sink compensation, thereby maintaining a favorable carbon balance. However, when carbon emissions exceed the sequestration capacity, it may lead to the depletion of soil organic carbon pools and the degradation of ecological functions, consequently posing potential threats to the ecological environment. This represents a key issue that warrants further investigation in the context of agricultural carbon sequestration and emission reduction. Therefore, following previous studies [10, 22], this study identifies carbon imbalance as the condition in which carbon emissions exceed carbon sequestration within a certain spatiotemporal scale, and quantifies the degree of imbalance in the carbon budget based on the relative difference between emissions and sequestration and its temporal variation. Detailed characterization methods are provided in Sect. "Calculation of carbon imbalance in agricultural cultivation.Calculation of carbon imbalance in agricultural cultivation".

### Calculation of carbon imbalance in agricultural cultivation

#### Calculation of carbon emissions from agricultural cultivation

Since the calculation of carbon imbalance in agricultural cultivation is based on the carbon budget, this paper first uses the emission factor method to calculate the carbon emissions generated by agricultural cultivation in each city in Hunan Province. Based on existing research findings and the agricultural cultivation conditions in the study area [25, 30], the agricultural cultivation carbon emissions in Hunan Province referred to in this paper mainly include CH_4_ emissions generated during rice growth, N_2_O emissions caused by the destruction of topsoil during the cultivation of rice, wheat, and corn, as well as carbon emissions resulting from the use of agricultural inputs in agricultural cultivation activities. The specific calculation formula (1) is as follows:

1 $$\begin{aligned}\:{C}_{E}=&\sum\!_{i=1}^{n}{T}_{i}\times\:{\delta\:}_{i}\times\:21+\sum\!_{j=1}^{n}{T}_{j}\\&\times\:{\delta\:}_{j}\times\:310+\sum\!_{k=1}^{n}{T}_{k}\times\:{\delta\:}_{k}\times\:\frac{44}{12}\end{aligned}$$

In the formula, $\:{C}_{E}$ represents the total carbon emissions generated by agricultural cultivation; $\:{T}_{i}$ represents the rice cultivation area for CH₄ emissions (including early rice, late rice, and medium rice); $\:{T}_{j}$ represents the grain crop cultivation area for N₂O emissions (including rice, wheat, and corn); $\:{T}_{k}$ represents the total amount of agricultural inputs used; $\:{\delta\:}_{i}$, $\:{\delta\:}_{j}$, and $\:{\delta\:}_{k}$ represent the emission factors for various carbon emission sources (Table 1); 21, 310, and 44/12 are the conversion factors for CH₄, N₂O, and C converted to CO₂ [31]. Since the statistical yearbook does not provide separate data on the amounts of agricultural inputs used in the growth of grain crops, this paper borrows the research method of Yang et al. to calculate the amount of various agricultural inputs used in grain production [32]. The calculation formula (2) is as follows:

2 $$\:{V}_{i}={P}_{i}\times\:\frac{{A}_{f}}{{A}_{c}}\:\text{}$$

In the formula, $\:{V}_{i}$ represents the amounts of agricultural inputs used for crop i during the cultivation of grain crops; $\:{P}_{i}$ represents the amounts of agricultural inputs used for crop i during the cultivation of crops; $\:{A}_{f}$ and $\:{A}_{c}$ represent the cultivation area of grain crops and the cultivation area of crops, respectively.

**Table 1:** Carbon emission sources from agricultural cultivation and their corresponding coefficients

Crop types	CH_4_ or *N*_2_O emission factors(kg/hm^2^)	References	Agricultural input	Carbon emission factor(kg/kg)	References
Early rice	147.100 (CH_4_) 0.240 (N_2_O)	[25]	Fertilizer	0.896	ORNL
Medium rice	562.800 (CH_4_) 0.240 (N_2_O)	[25]	Pesticide	4.934	ORNL
Late rice	341.000 (CH_4_) 0.240 (N_2_O)	[25]	Agricultural film	5.180	IREENAU
Wheat	2.050 (N_2_O)	[33]	Diesel fuel	0.593	IPCC
Corn	2.530 (N_2_O)	[33]	Irrigation	266.480	[34]

The unit of carbon emission coefficient for irrigation in the above table is kg/hm^2^. In addition, ORNL and IREENAU stand for Oak Ridge National Laboratory and the Institute of Agricultural Resources and Environmental Sciences, Nanjing Agricultural University, respectively

#### Calculation of carbon sequestration in agricultural cultivation

Carbon sequestration in agricultural cultivation mainly includes two parts: carbon absorbed from the atmosphere by crops through photosynthesis and carbon fixed in farmland soil. The specific calculation formula (3) is as follows [30]:

3 $$\:{C}_{A}=\sum\:_{i=1}^{n}{c}_{i}\times\:{Y}_{i}\times\:\frac{1-r}{{HI}_{i}}+{F}_{S}\times\:A$$

Among these, $\:{C}_{A}$ represents the carbon sequestration during agricultural cultivation; $\:n$ denotes the type of grain crop; $\:{c}_{i}$ represents the carbon sequestration rate of the grain crop; $\:{Y}_{i}$ and $\:{HI}_{i}$ represent the economic yield and economic coefficient of the grain crop, respectively; $\:r$ and $\:{F}_{S}$ represent the moisture content of the grain crop and the carbon sequestration rate of farmland soil, respectively, with the carbon sequestration rate set at 0.892 t/(hm²·a⁻¹) [35]; $\:A$ is the cultivated land area. The correlation coefficients for carbon sequestration of various grain crops are shown in Table 2 [36].

**Table 2:** Calculate the correlation coefficients for carbon sequestration by various grain crops

Grain crops	Economic coefficient	Moisture content	Carbon sequestration rate
Rice	0.450	0.120	0.414
Wheat	0.400	0.120	0.485
Corn	0.400	0.130	0.471

#### Calculation of carbon imbalance in agricultural cultivation

Based on the aforementioned definition of agricultural carbon imbalance and in conjunction with existing research findings [10, 22], the formula (4) used in this paper to calculate the agricultural carbon imbalance of each city in Hunan Province is as follows:

4 $$\begin{aligned}\:{CII}_{i}&=\alpha\:\times\:\left|\frac{{CE}_{i}/\mathrm{C}\mathrm{E}{-CA}_{i}/CA}{{CA}_{i}/CA}\right|+\beta\\&\:\times\:\left[\left|\frac{\left({CE}_{i}^{t}-{CE}_{i}^{0}\right)}{{CE}_{i}^{0}}\right|-\left|\frac{\left({CA}_{i}^{t}-{CA}_{i}^{0}\right)}{{CA}_{i}^{0}}\right|\right]\end{aligned}$$

In the formula, $\:{CII}_{i}$ represents the carbon imbalance index; $\:\alpha\:$ and $\:\beta\:$ are weights determined using the entropy method, with values of 0.656 and 0.344, respectively; $\:{CE}_{i}$ and $\:{CA}_{i}$ represent the carbon emissions and carbon sequestration of each city in Hunan Province, respectively; $\:CE$ and $\:CA$ represent the total carbon emissions and total carbon sequestration of Hunan Province, respectively; $\:{CE}_{i}^{0}$ and $\:{CA}_{i}^{0}$ are the carbon emissions and carbon sequestration of the city region in the initial year; $\:{CE}_{i}^{t}$and $\:{CA}_{i}^{t}$ are the carbon emissions and carbon sequestration of the city region in the final year. In addition, to facilitate comparisons of the CII across different years and city units, this paper adopts the extreme value normalization method to process the agricultural cultivation CII of each city unit in Hunan Province.

### Exploratory spatial-temporal data analysis (ESTDA)

#### Morlan index

To explore whether there are overall correlations and local clustering characteristics in the spatial distribution of CII in agricultural cultivation in Hunan Province, this paper adopts the global Moran index ($\:{I}_{g}$) and local Moran index ($\:{I}_{i}$) to measure it [37]. Formulas 5 and 6 are presented as follows:

5 $$\:{I}_{g}=\frac{n\sum\:_{i=1}^{n}\sum\:_{j=1}^{n}{w}_{i\mathrm{j}}\left({x}_{i}-\stackrel{-}{x}\right)\left({x}_{j}-\stackrel{-}{x}\right)}{\sum\:_{i=1}^{n}\sum\:_{j=1}^{n}{w}_{ij}\sum\:_{i=1}^{n}{\left({x}_{i}-\stackrel{-}{x}\right)}^{2}}$$

6 $$\:{I}_{i}=\frac{n\left({x}_{i}-\stackrel{-}{x}\right)\sum\:_{j=1}^{n}{w}_{ij}\left({x}_{j}-\stackrel{-}{x}\right)}{\sum\:_{i=1}^{n}{\left({x}_{i}-\stackrel{-}{x}\right)}^{2}}$$

In the formula, the values of $\:{I}_{g}$ and $\:{I}_{i}$ are both in the range of [−1, 1]. $\:{I}_{g}$>0 indicates that the CII in the study area is positively correlated overall, $\:{I}_{g}$<0 indicates that it is negatively correlated overall, and $\:{I}_{g}$=0 indicates that it is not correlated overall. $\:{I}_{i}$>0 indicates that the CII of a spatial unit is high-high or low-low aggregated with that of its neighboring spatial units. $\:{I}_{i}$<0 indicates that CII exhibits high-low or low-high clustering in local space; $\:{x}_{i}$ and $\:{x}_{j}$ are the CII of spatial units $\:i$ and $\:j$, respectively; $\:\stackrel{-}{x}$ is the average CII value of the study area; $\:{w}_{ij}$ is the weight of spatial units $\:i$ and $\:j$; and $\:n$ is the number of spatial units.

#### LISA time path

The LISA (local indicators of spatial association) time path can characterize the changing trends and stability of positional shifts of the agricultural cultivation CII in Hunan Province within Moran’s I scatter plot, thereby reflecting the dynamic changes of the CII in local spatial contexts [38]. The formulas for calculating the relative length and curvature in the LISA time path are given in formulas 7 and 8, respectively [39].

7 $$\:{d}_{i}=\frac{n\times\:\sum\:_{t=1}^{T-1}d\left({L}_{i,t},{L}_{i,t+1}\right)}{\sum\:_{i=1}^{n}\sum\:_{t=1}^{T-1}d\left({L}_{i,t},{L}_{i,t+1}\right)}$$

8 $$\:{f}_{i}=\frac{\sum\:_{t=1}^{T-1}d\left({L}_{i,t},{L}_{i,t+1}\right)}{d\left({L}_{i,1},{L}_{i,T}\right)}$$

In the formula, $\:{d}_{i}$ represents the relative length; $\:{f}_{i}$ represents the curvature; $\:n$ denotes the number of city areas; $\:T$ stands for the time interval; $\:{L}_{i,t}$ indicates the position coordinates of city area $\:i$ in the Moran’s I scatter plot in year $\:t$; $\:d\left({L}_{i,t},\:{L}_{i,t+1}\right)$ refers to the movement distance of city area$\:i$ from year$\:t$ to year $\:t+1$; and $\:d\left({L}_{i,1},{L}_{i,T}\right)$ is the distance traveled by city area $\:i$ from the starting year to the end of the study period.

#### Standard deviational ellipse (SDE)

SDE is a classic method in spatial statistical analysis, used to describe the spatial distribution characteristics of geographical elements. This paper employs the SDE method to analyze the migration trajectory of the center of gravity of the agricultural cultivation CII in Hunan Province. The calculation formulas for SDE-related parameters are given as follows [40]:

9 $$\:\left(\overline{x}, \overline{y}\right)=\left(\frac{\sum\!_{i=1}^{n}{w}_{i}{x}_{i}}{\sum\!_{i=1}^{n}{w}_{i}}, \frac{\sum\!_{i=1}^{n}{w}_{i}{y}_{i}}{\sum\!_{i=1}^{n}{w}_{i}}\right)$$

10 $$\:{tan}\theta\:=\frac{\left(\sum\:_{i=1}^{n}{w}_{i}^{2}{\stackrel{-}{x}}_{i}^{2}-\sum\:_{i=1}^{n}{w}_{i}^{2}{\stackrel{-}{y}}_{i}^{2}\right)+\sqrt{{(\sum\:_{i=1}^{n}{w}_{i}^{2}{\stackrel{-}{x}}_{i}^{2}-\sum\:_{i=1}^{n}{w}_{i}^{2}{\stackrel{-}{y}}_{i}^{2})}^{2}-4\sum\:_{i=1}^{n}{w}_{i}^{2}{\stackrel{-}{x}}_{i}^{2}{\stackrel{-}{y}}_{i}^{2}}}{2{\sum\:}_{i=1}^{n}{w}_{i}^{2}{\stackrel{-}{x}}_{i}{\stackrel{-}{y}}_{i}}$$

11 $$\:{\:\sigma\:}_{x}=\sqrt{\frac{\sum\:_{i=1}^{n}{\left({w}_{i}{\stackrel{-}{x}}_{i}{cos}\theta\:-{w}_{i}{\stackrel{-}{y}}_{i}{sin}\theta\:\right)}^{2}}{\sum\:_{i=1}^{n}{w}_{i}^{2}}}\:,\:{\sigma\:}_{y}=\sqrt{\frac{\sum\:_{i=1}^{n}{\left({w}_{i}{\stackrel{-}{x}}_{i}{sin}\theta\:-{w}_{i}{\stackrel{-}{y}}_{i}{cos}\theta\:\right)}^{2}}{\sum\:_{i=1}^{n}{w}_{i}^{2}}}$$

In the formula, $\:\left(\overline{x}, \overline{y}\right)$ represents the coordinates of the center of gravity, indicating the relative spatial distribution of the agricultural cultivation CII in Hunan Province; $\:{x}_{i}$ and $\:{y}_{i}$ denote the central coordinates of each city unit within the study area; $\:{w}_{i}$ represents the weight of the study unit; $\:\theta\:$ is the elliptical azimuth, reflecting the trending direction of the CII distribution; $\:{\stackrel{-}{x}}_{i}$ and $\:{\stackrel{-}{y}}_{i}$ represent the deviations of the central coordinates of the study unit from the center of gravity; $\:{\:\sigma\:}_{x}$ and $\:{\sigma\:}_{y}$ are the standard deviations along the major and minor axes, respectively, reflecting the direction and density of changes in the agricultural cultivation CII.

### **PLS-VIP method**

The partial least squares variable importance projection (PLS-VIP) method can accurately identify the degree of importance of explanatory variables on target variables by calculating the VIP values of variables, and can effectively eliminate collinearity between variables [41]. Based on the characteristics of agricultural cultivation activities and in conjunction with existing research findings [42], this paper takes the agricultural cultivation CII in Hunan Province as the dependent variable, and selects corresponding indicators from five dimensions—production input, land use, policy intervention, economic factors, and natural conditions—as independent variables (Table 3). Finally, the PLS-VIP method is employed to identify the importance of these influencing factors.

For the independent variable $\:{x}_{j}$, the formula for calculating its $\:{vip}_{j}$ value is:

**Table 3:** Indicator system for driving factors of CII in agricultural cultivation

Dimension	Index	Full name of indicator	Indicator source or explanation
Production inputs	FUI	Fertilizer use intensity	Fertilizer usage/grain planting area
	PUI	Pesticide use intensity	Pesticide usage/grain planting area
	IAF	Intensity of agricultural film use	Agricultural film usage/grain planting area
	PAMP	Per capita agricultural machinery power	Total agricultural machinery power/agricultural population
Land use	GPA	Grain planting area	《Hunan Province Rural Statistical Yearbook》
	GDR	Grain disaster rate	Area affected by grain disasters/grain planting area × 100%
Policy intervention	IARDF	Investment in agricultural R&D funds	Internal expenditure on R&D × (total agricultural output/regional GDP)
	FSA	Financial support for agriculture	Local government agricultural budget expenditure/total local government budget expenditure × 100%
Economic factors	PAOV	Per capita agricultural output value	Total agricultural output/agricultural population
Natural conditions	GST	Growing season temperature	《Hunan Statistical Yearbook》
	GSP	Growing season precipitation	《Hunan Statistical Yearbook》

12 $$\:{vip}_{j}=\sqrt{m{\sum\:}_{h=1}^{k}{\omega\:}_{hj}^{2}{r}_{h}^{2}\left(Y;{t}_{h}\right)/{\sum\:}_{h=1}^{k}{r}_{h}^{2}\left(Y;{t}_{h}\right)}$$

In the equation, $\:m$ denotes the number of independent variables; $\:k$ represents the number of extracted latent variables; $\:{\omega\:}_{hj}$ is the weight of independent variable $\:{x}_{j}$ on latent variable $\:{t}_{h}$, reflecting the marginal contribution of $\:{x}_{j}$ to $\:{t}_{h}$, with $\:{\sum\:}_{j=1}^{m}{\omega\:}_{hj}^{2}={\omega\:}_{h}^{T}{\omega\:}_{h}=1;{r}_{h}\left(Y;{t}_{h}\right)$ stands for the correlation coefficient between dependent variable $\:Y$ and the score vector $\:{t}_{h}$ of the $\:h$-th latent variable, where $\:{r}_{h}\left(Y,{t}_{h}\right)={u}_{h}^{T}{t}_{h}$. Specifically, if $\:{vip}_{j}$ >1, $\:{x}_{j}$ is an important driving factor of the dependent variable; if 0.8<$\:{vip}_{j}$<1, $\:{x}_{j}$ is a relatively important driving factor; and if $\:{vip}_{j}$<0.8, $\:{x}_{j}$ is considered an unimportant factor.

### Geographically and temporally weighted regression (GTWR)

GTWR is an extension of geographic weighted regression (GWR) that introduces a time dimension and comprehensively considers spatiotemporal non-stationarity to perform local regression on variables. This method helps, to some extent, reduce parameter estimation bias and model errors, thereby enhancing the robustness of regression results [43, 44]. To investigate whether there is regional heterogeneity in the influencing factors of CII in agricultural cultivation across cities in Hunan Province from 2001 to 2022, this paper uses the GTWR model for analysis. The model formula 13 is as follows:

13 $$\begin{aligned}\:{CII}_{i}=&{\beta\:}_{0}\left({u}_{i},{v}_{i},{t}_{i}\right)\\&+{\sum\:}_{k=1}^{n}{\beta\:}_{k}\left({u}_{i},{v}_{i},{t}_{i}\right){X}_{ik}+{\epsilon\:}_{i}\end{aligned}$$

In the equation, $\:{CII}_{i}$ denotes the agricultural cultivation carbon imbalance index for city $\:i$; $\:\left({u}_{i},{v}_{i},{t}_{i}\right)$ represents the latitude, longitude, and time corresponding to city $\:i$; $\:{\beta\:}_{0}\left({u}_{i},{v}_{i},{t}_{i}\right)$ is a constant term; $\:{\beta\:}_{k}\left({u}_{i},{v}_{i},{t}_{i}\right)$ stands for the regression coefficient of the $\:k$-th variable in city $\:i$, reflecting its degree of influence on the agricultural cultivation CII; $\:{X}_{ik}$ is the $\:k$-th independent variable for city $\:i$; $\:n$ indicates the number of independent variables; and $\:{\epsilon\:}_{i}$ is the random error term, which follows an independent and identically distributed *N*(0, *σ*^2^) distribution.

### **Data source**

The raw data for calculating the carbon budget of agricultural cultivation—including grain cultivation area, grain crop yield, agricultural input usage, and effective irrigation area—are all derived from the Hunan Rural Statistical Yearbook, Hunan Statistical Yearbook, statistical yearbooks of various cities in Hunan Province, and statistical bulletins, all covering the period from 2001 to 2023. For partially missing data in individual city areas, interpolation methods were employed for supplementation. In addition, all vector data used in this article are sourced from the National Geographic Information Resource Directory Service System (https://www.webmap.cn).

## Results and analysis

### **Time variation in agricultural cultivation CII**

**Fig. 2:**
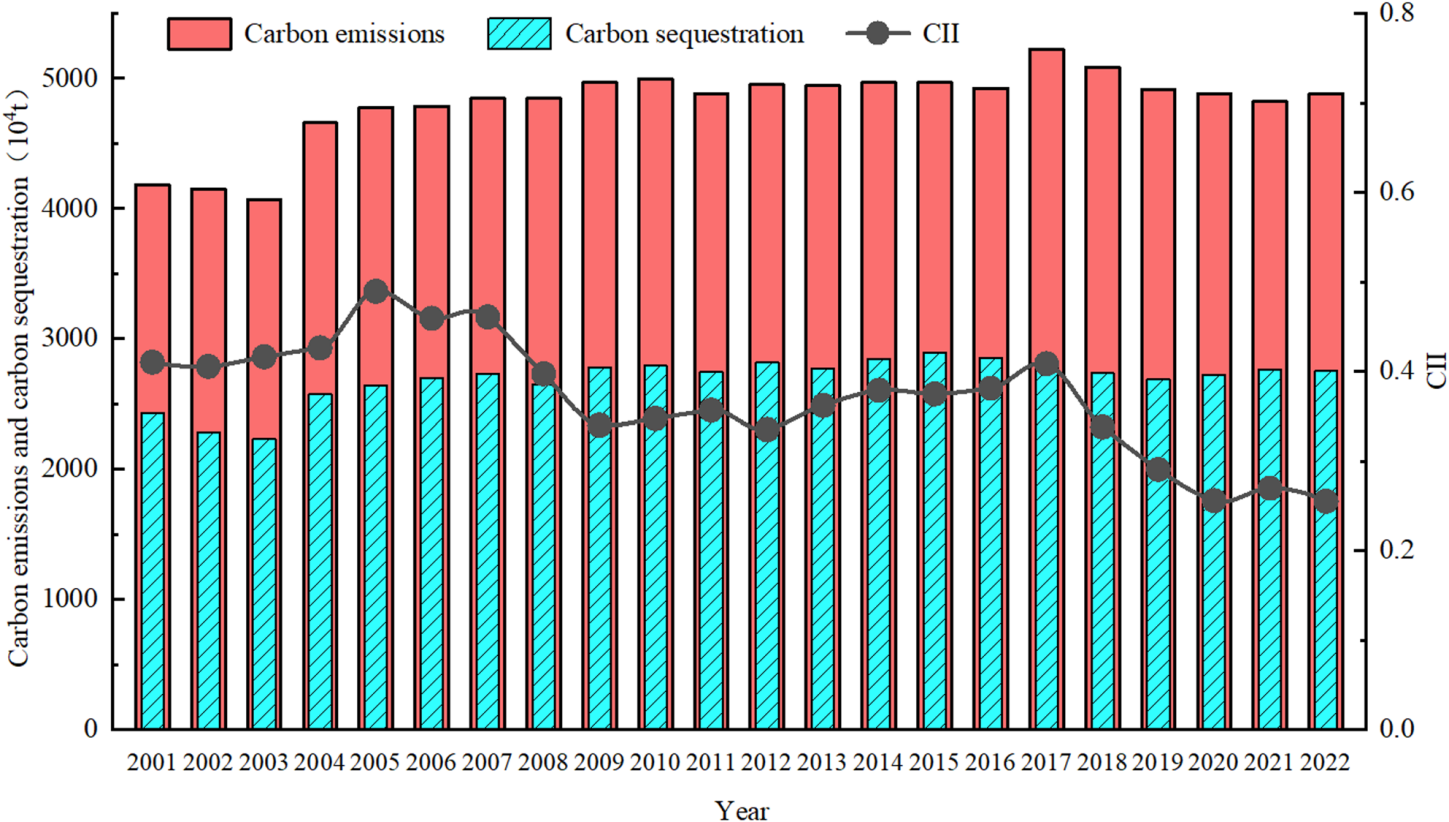
Temporal changes in carbon emissions, carbon sequestration, and CII from agricultural cultivation in Hunan Province

From the changes in agricultural cultivation-related carbon emissions, carbon sequestration, and CII in Hunan Province (Fig. 2), carbon sequestration exhibited a fluctuating growth trend between 2001 and 2022. However, carbon emissions remained substantially higher than carbon sequestration, thus maintaining a persistent state of carbon imbalance in the province’s agricultural cultivation. Over the same period, the CII showed an overall downward trend. Specifically, between 2001 and 2009, the CII reached its maximum value of 0.49 in 2005 before beginning to decline. This trend may be attributed to such measures as China’s increased direct subsidies to grain farmers starting in 2005 and the complete abolition of traditional agricultural taxes in 2006 [45]. The above policies boosted farmers’ enthusiasm for grain production and facilitated large-scale agricultural development, thereby alleviating the CII. The maximum CII value (0.41) occurred in 2017 during 2009–2022. Research findings indicate that carbon emissions from agricultural cultivation in Hunan Province peaked at 52.2588 million tons in 2017, whereas carbon sequestration amounted to only 27.9934 million tons, resulting in a carbon emission-to-sequestration ratio of 1.87. The primary reason may be that the cultivation areas of early rice and late rice decreased in Hunan Province in 2017, whereas the cultivation area of medium rice increased by approximately 5.54 × 10⁵ hm² compared to the previous year. Unlike early and late rice, the emission coefficient of medium rice reaches 562.8 kg/hm² [25, 33]. Therefore, the high value of the CII in 2017 may be closely associated with the expansion of medium rice cultivation areas that year. After 2017, the gap in the carbon budget of agricultural cultivation gradually narrowed, and the CII dropped rapidly, which indicates that the state of carbon imbalance in agricultural cultivation in Hunan Province is showing a positive trend.

**Fig. 3:**
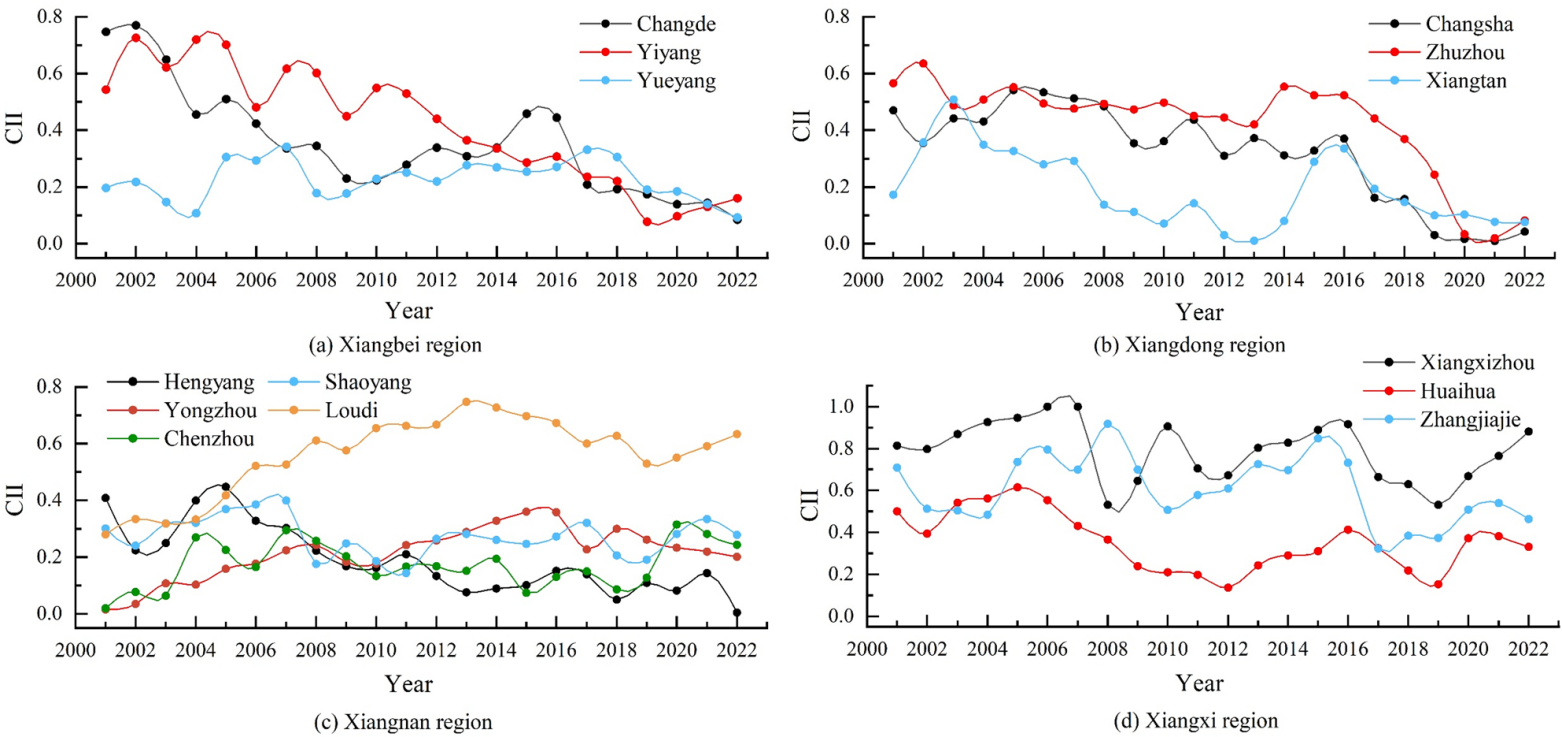
Temporal changes of agricultural cultivation CII in each city

At the city scale, the CII for agricultural cultivation in various cities of Hunan Province exhibited different evolutionary trends over time from 2001 to 2022 (Fig. 3). During the study period, the CII in Changde and Yiyang, located in the Xiangbei region, fluctuated significantly, with a range from 0.08 to 0.77(Fig. 3a). The maximum values for both cities occurred in 2002, while the minimum values were recorded in 2019 (Yiyang) and 2022 (Changde), respectively. Although the CII of Changde and Yiyang has shown a fluctuating downward trend since 2002, Changde’s CII reached a secondary peak during 2015–2016. The primary reason for this is that during this period, an expansion in the area dedicated to rice cultivation in Changde led to a significant increase in agricultural inputs (e.g., the usage of chemical fertilizers and agricultural film in 2015 increased by approximately 3% and 14%, respectively, compared to 2014). This increase in agricultural inputs resulted in carbon emissions growing at a rate significantly faster than that of carbon sequestration over the same period, thereby causing a rapid rise in the CII. After reaching its minimum in 2019, the CII for agricultural cultivation in Yiyang has gradually increased, which may be associated with the expansion of the rice cultivation area and increased fertilizer usage in Yiyang since 2019. According to statistics, the rice cultivation area in Yiyang in 2020 increased by approximately 1.7 × 10⁴ hm² compared to 2019, with a corresponding increase in fertilizer usage of about 8%—the phenomenon that contributed to the rise in CII. The changes in the CII of agricultural cultivation in Yueyang are relatively small, generally fluctuating within the range of 0.09–0.34. Overall, since 2017, the fluctuation range of CII across cities in the Xiangbei region has begun to narrow, indicating that the differences in carbon imbalance among these cities are gradually diminishing. Figure 3b showed that the CII of agricultural cultivation in the Xiangdong region exhibited fluctuations before 2016. Among these cities, Xiangtan experienced the most significant fluctuations, which may be associated with substantial variations in agricultural inputs, arable land area, and the level of agricultural mechanization within the city prior to 2016. After 2016, the CII in the Xiangdong region declined rapidly, possibly due to such measures as the active promotion of “rice-shrimp co-cultivation” and the reduction of the medium-season rice planting area in the region [46]. These measures led to a year-on-year decrease in the growth rate of carbon emissions from agricultural cultivation in the Xiangdong region, resulting in a significant reduction in the CII of each city region, which reached its lowest value in 2021 (Fig. 3b). In the Xiangnan region, except for Loudi and Yongzhou, where the CII showed a fluctuating upward trend, the CII in other cities exhibited an “M”-shaped fluctuation pattern (Fig. 3c). Taking Yongzhou as an example, from 2001 to 2016, its agricultural cultivation activities continued to rely on high-carbon-emission agricultural machinery inputs (e.g., the total power of agricultural machinery grew at an average annual rate of 8.5% during this period), leading to a faster growth rate of carbon emissions than that of carbon sequestration over the same period, thereby causing a continuous rise in the CII. After 2016, however, due to fluctuating reductions in rice cultivation area and agricultural inputs in Yongzhou, the growth rate of carbon emissions declined, which in turn resulted in a decrease in its CII. The rising trend of agricultural cultivation of CII in Loudi may be attributed to the expansion of the city’s medium rice cultivation area. Although driven by the dual reduction policy for pesticides and fertilizers,” the usage of chemical fertilizers and pesticides in Loudi decreased by 25.9% and 39.1%, respectively, in 2022 compared to 2016, and the area under medium rice cultivation increased from 18.43 × 10³ hm² in 2001 to 63.15 × 10³ hm² in 2022. Given that the CH₄ emission factor for medium rice is significantly higher than that for early and late rice, this has led to a notable upward trend in Loudi’s CII. In the Xiangxi region, the CII of Xiangxizhou and Zhangjiajie exhibits an ‘M-shaped’ fluctuation pattern, but its long-term trend is not significant (Fig. 3d). This may be attributed to the predominantly mountainous and hilly terrain, which results in fragmented cropland and, to some extent, constrains the large-scale dissemination and application of low-carbon agricultural technologies [29]. Meanwhile, the well-developed tourism sector in these areas has driven frequent conversions between tourism land and agricultural land, undermining the stability of carbon sources and sinks in agricultural cultivation and thereby exacerbating stage-wise fluctuations in CII. By contrast, Huaihua shows relatively moderate CII fluctuations, with higher values mainly concentrated before 2009. In addition, some farmers in the Xiangxi region have limited awareness of low-carbon production, and the transition from traditional high-carbon farming practices has been slow. This may also be an important factor contributing to the persistently high CII values observed in agricultural cultivation across most cities within the region [25].

### **Spatial pattern characteristics of agricultural cultivation CII**

#### Spatial variation of CII

**Fig. 4:**
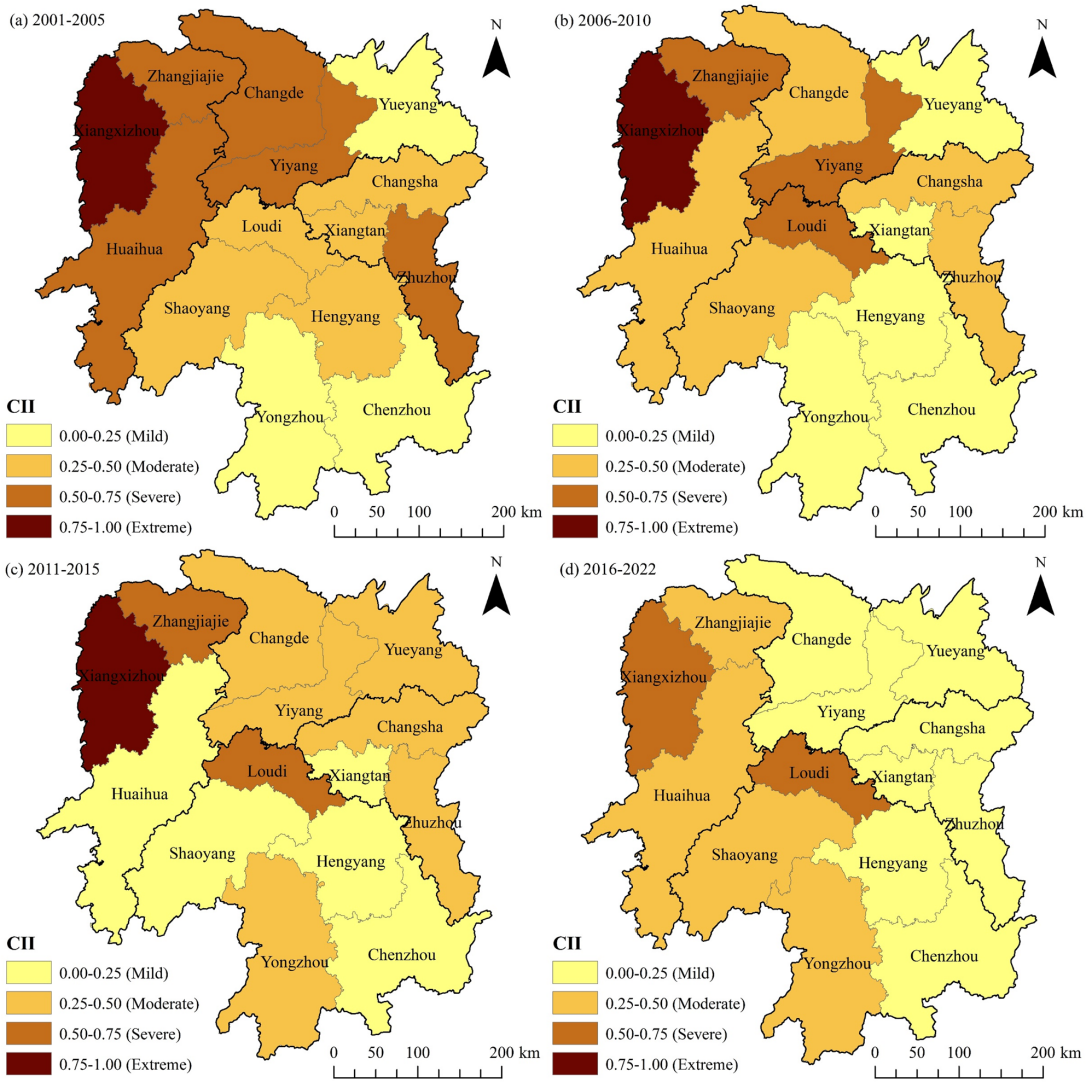
Spatial distribution of CII for agricultural cultivation across cities in Hunan Province (CII in the figure represents the average value for each city over the study period)

To reveal the spatial variation characteristics of the CII in agricultural cultivation in Hunan Province, this paper employs the natural breaks method in ArcGIS 10.8 software, combined with manual adjustment of breakpoints, to classify the CII of each city into four levels: mild carbon imbalance (0.00–0.25.00.25), moderate carbon imbalance (0.25–0.50), severe carbon imbalance (0.50–0.75), and extreme carbon imbalance (0.75–1.00.75.00). Figure 4 shows that from 2001 to 2022, the spatial distribution of the agricultural cultivation CII in Hunan Province gradually shifted from “high in the north and low in the south” to “high in the west and low in the east.” The area of mild carbon imbalance gradually expanded, while areas with moderate carbon imbalance underwent a spatial transformation from the southwest to the northeast and then back to the southwest. Meanwhile, areas with severe carbon imbalance exhibited a decreasing trend over time. From 2001 to 2005, except for Xiangxizhou, which was in a state of extreme carbon imbalance, 10 prefecture-level cities in the province were in a state of moderate to severe carbon imbalance, accounting for approximately 71% of the total number of prefecture-level cities in the province; the remaining ones—Yueyang, Yongzhou, and Chenzhou—were all in a state of mild carbon imbalance (Fig. 4a). Compared with 2001–2005, Xiangxizhou remained in a state of extreme carbon imbalance during 2006–2010, but the number of prefecture-level cities with severe carbon imbalance decreased from five to three. Specifically, Changde, Huaihua, and Zhuzhou, which were previously in a state of severe carbon imbalance, transitioned to moderate carbon imbalance; meanwhile, Xiangtan and Hengyang shifted from moderate to mild carbon imbalance (Fig. 4b). The increase in cities with mild and moderate carbon imbalance may be attributed to Hunan Province’s vigorous development of resource-saving agriculture during the “Eleventh Five-Year Plan” period (2006–2010). The comprehensive promotion of actions for reducing chemical fertilizers and pesticides and improving their efficiency significantly enhanced the utilization efficiency of agricultural materials, resulting in a substantial decline in the growth rate of carbon emissions from agricultural cultivation. Furthermore, the improved level of agricultural mechanization in townships, which reduced equipment energy consumption, may also be a non-negligible contributing factor [47]. The shift in Loudi from a moderate carbon imbalance during 2001–2005 to a severe one during 2006–2010 may be attributed to the increased use of agricultural film and the growth in total power of agricultural machinery, which accelerated the growth rate of carbon emissions. Studies indicate that in Loudi, the usage of agricultural film and the total power of agricultural machinery increased from 964.11 tons and 1.47 × 10⁶ kW in 2006 to 1,407.74 tons and 1.93 × 10⁶ kW in 2010, respectively. Figure 4c shows that, from 2011 to 2015, areas with moderate carbon imbalance in agricultural cultivation across Hunan Province exhibited a shifting trend from the southwest to the northeast compared to the previous period. This trend was likely due to the annual increase in rice cultivation areas in the regions of northern and eastern Hunan during this period, which led to a corresponding rise in agricultural inputs and, consequently, an increase in carbon emissions. Since carbon sequestration did not increase synchronously, the CII generally rose in these regions. However, Xiangxizhou, Zhangjiajie, and Loudi remain in extremely or severely carbon-imbalanced zones, indicating that their carbon imbalance status has not improved significantly and that they face a high risk of such imbalance. From 2016 to 2022, cities with extreme carbon imbalance in Hunan Province disappeared. Except for Xiangxizhou and Loudi, which remained severely carbon-imbalanced, all other cities were in a state of mild or moderate carbon imbalance, marking a significant improvement in agricultural cultivation carbon imbalance compared to the previous period (Fig. 4d). This improvement may be attributed to the “Implementation Opinions on Innovating Institutional Mechanisms to Promote Green Agricultural Development” issued by the state in 2017. This initiative spurred Hunan Province to promote green agriculture actively, leading to a substantial reduction in carbon emissions from agricultural practices and a marked decrease in the CII. As a result, many cities in Hunan Province have made significant strides in agricultural carbon sequestration and emission reduction. Notably, throughout the study period, agricultural cultivation in Xiangxizhou remained in a state of extremely or severely carbon imbalance, which is potentially linked to the high degree of cultivated land fragmentation in the region. Generally, cultivated land fragmentation compromises the stability of arable land, leading to soil degradation and reduced carbon storage, thereby exacerbating carbon imbalance in agricultural cultivation and driving up the CII [6, 48]. This also signifies that Xiangxizhou faces a long-term challenge in carbon sequestration and emission reduction for agricultural cultivation. In Loudi, from 2006 to 2022, heavy reliance on agricultural machinery resulted in a sustained increase in fuel consumption and total agricultural machinery power, with annual growth rates of 1.42% and 3.45%, respectively. Concurrently, from 2015 to 2022, accelerated industrialization in Loudi led to a reduction of nearly 33,270 hectares in grain cultivation area, causing a significant decline in carbon sequestration capacity. The combined effect of these two factors kept Loudi’s CII in a state of severe carbon imbalance during this period.

**Fig. 5:**
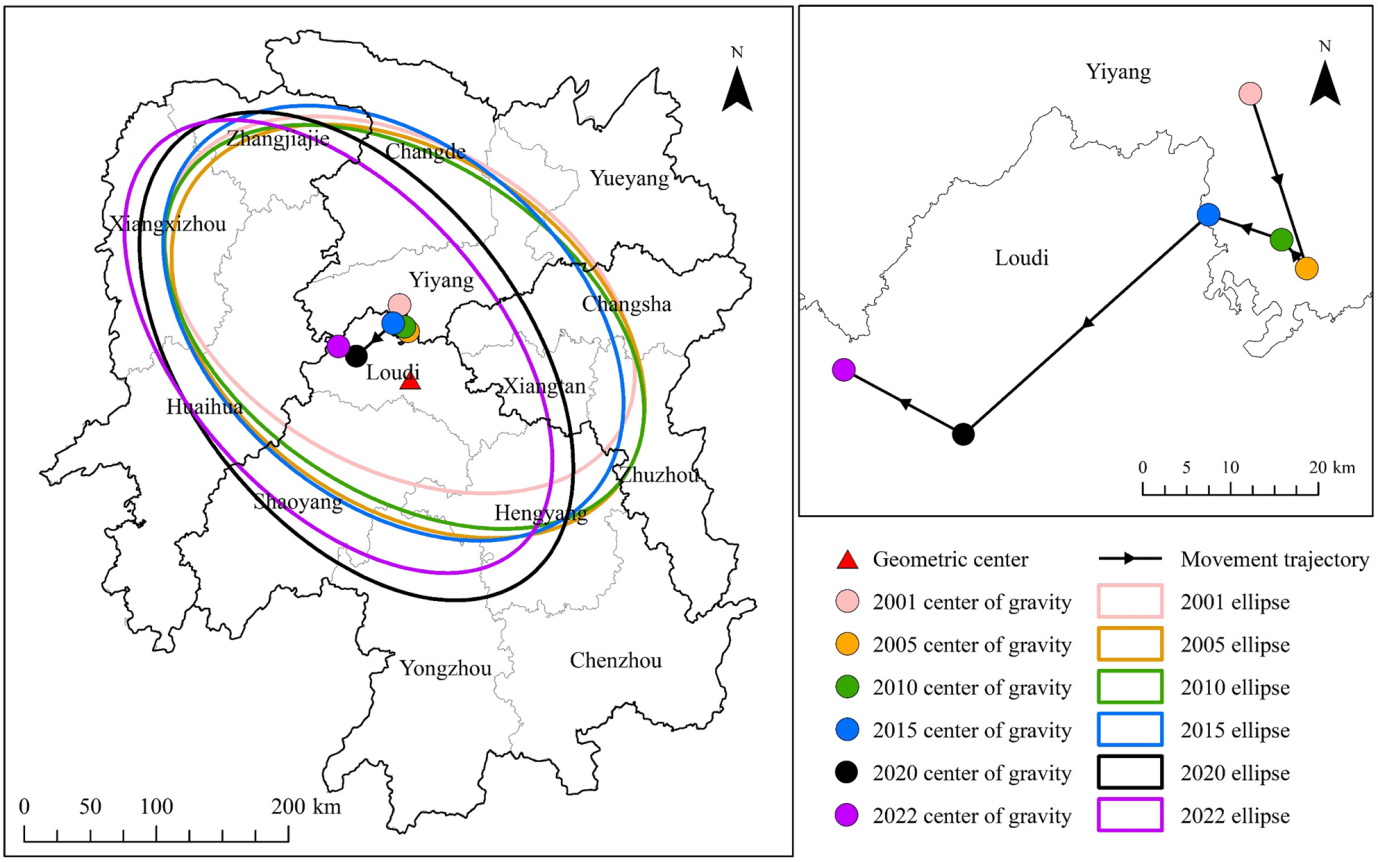
Standard deviation ellipse and migration path of the gravity center of CII in agricultural cultivation across Hunan Province

To further explore the trajectory of the CII’s gravity center migration, this paper employs the standard deviation ellipse method to analyze the evolutionary characteristics of the gravity center of agricultural cultivation CII in Hunan Province from 2001 to 2022. As shown in Fig. 5, the CII’s gravity center is located northwest of Hunan Province’s geometric center, generally moving from northeast to southwest. It gradually shifted from Yiyang to Loudi, with its location ranging between 111.06°E–111.60°E and 27.90°N–28.25°N. This is consistent with the spatial distribution pattern of agricultural cultivation CII across Hunan Province’s city areas, as depicted in Fig. 4 and discussed earlier. Based on the elliptical parameters (Table 4), the ellipse circumference increased from 1002.83 km to 1030.65 km, and its area expanded from 74456.45 km² to 75374.12 km² between 2001 and 2022. This indicates that the spatial dispersion range of agricultural cultivation CII in Hunan Province expanded during the study period. Additionally, the semi-major and semi-minor axes varied within the ranges of 192.06–212.01 km and 117.05–133.85 km, respectively, while the ellipse’s rotation angle increased from 119.22° to 138.24° over the same period. These changes suggest that both the spatial dispersion range and orientation of agricultural cultivation CII in Hunan Province are continuously evolving, potentially due to the combined effects of factors such as adjustments in agricultural cultivation structures, changes in arable land area, agricultural input levels, and improvements in agricultural management practices across various city regions during the study period.

**Table 4:** Standard deviation ellipse parameters of CII for agricultural cultivation in Hunan Province

Year	Coordinates		Elliptical half axis		Rotation angle (°)	Circumference (km)	Area (km^2^)	Location
	Longitude (°)	Latitude (°)	Long (km)	Short (km)				
2001	111.536	28.252	192.064	123.406	119.216	1002.827	74456.445	Yiyang
2005	111.600	28.071	197.508	132.841	124.605	1047.765	82420.765	Yiyang
2010	111.571	28.101	201.507	125.576	123.692	1041.429	79490.727	Yiyang
2015	111.486	28.127	199.002	133.846	130.712	1055.694	83672.806	Yiyang
2020	111.201	27.901	212.006	127.408	142.281	1082.902	84852.425	Loudi
2022	111.063	27.968	204.994	117.048	138.235	1030.651	75374.117	Loudi

#### Spatial correlation characteristics of CII

**Fig. 6:**
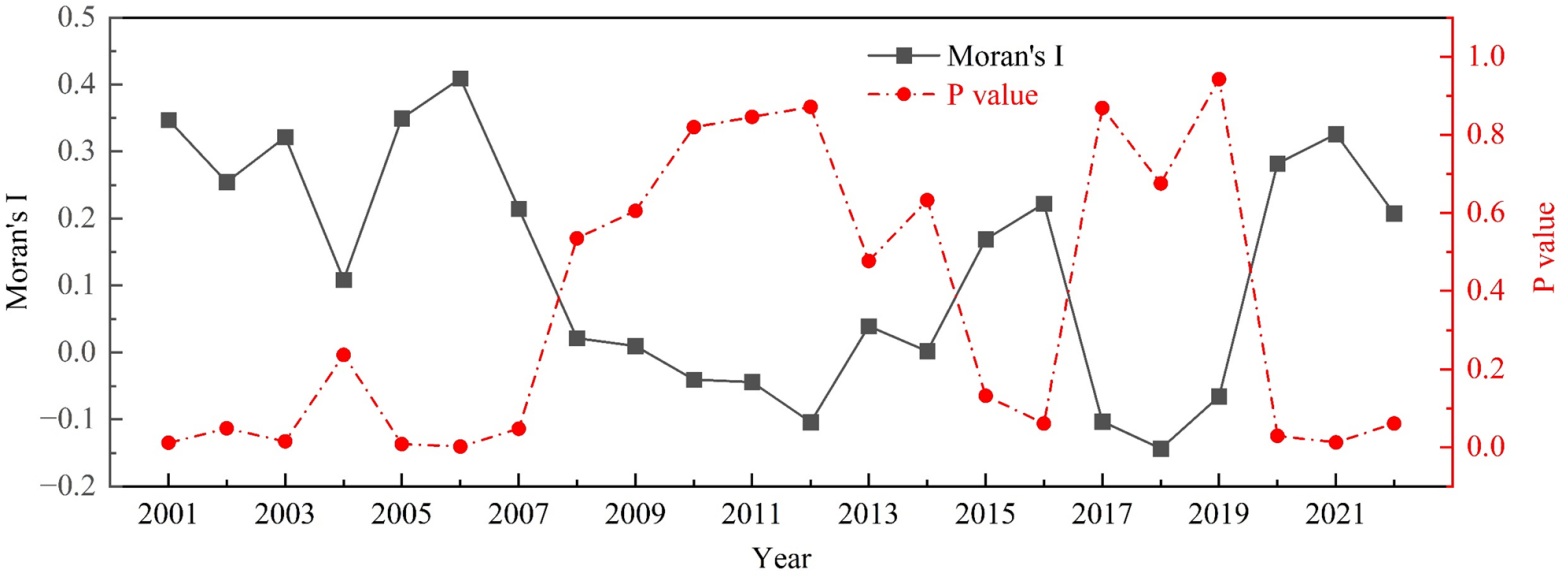
Temporal changes in the global Moran’s I of CII for agricultural cultivation in Hunan Province

Figure 6 shows that the global Moran’s I of agricultural cultivation CII in Hunan Province fluctuated between 2001 and 2022, without exhibiting an obvious trend. Specifically, from 2001 to 2007, the global Moran’s I ranged from 0.108 to 0.409, with all values (except 2004) passing the significance test at the 5% level, indicating a significant positive spatial correlation of agricultural cultivation CII in Hunan Province during this period. The primary reason for this lies in the fact that between 2001 and 2007, the state increased direct subsidies to grain farmers, while the full abolition of agricultural taxes further stimulated their enthusiasm for grain production [50]. To boost agricultural income, farmers across city regions significantly increased their input of agricultural materials, leading to a synchronous regional rise in agricultural carbon emissions in Hunan Province and consequently a significant positive spatial correlation of CII. From 2008 to 2019, the global Moran’s I of CII ranged from − 0.144 to 0.222, exhibiting an overall fluctuating trend of first decreasing, then increasing, and then decreasing again. However, none of these values were statistically significant. This indicates that the spatial correlation of CII during this period showed a fluctuating weakening trend, with its overall spatial association characteristics approaching a random distribution. This phenomenon may be attributed to discrepancies in the implementation of a series of national green agriculture policies across cities in Hunan Province during this period. For instance, the No. 1 Central Documents of 2008 and 2014 explicitly proposed vigorously developing resource-saving and eco-friendly agriculture, while those of 2016 and 2017 further emphasized promoting sustainable and green agricultural development [45, 50]. Although Hunan Province concurrently implemented a series of emission reduction and carbon sequestration measures—such as low-carbon agriculture pilot projects and the promotion of diversified cultivation structures—variations in policy enforcement intensity and technology promotion effectiveness across city regions led to significant emission reduction outcomes in some areas, while others continued to rely on traditional high-carbon production models. Consequently, the spatial coordination of CII in agricultural cultivation in Hunan Province was disrupted, resulting in weakened correlation. From 2020 to 2022, the spatial positive correlation of CII exhibited fluctuations. Specifically, in 2020 and 2021, Moran’s I returned to positive values, and both passed the significance test; however, in 2022, the positive correlation weakened slightly and failed the significance test. This indicates that the spatial association characteristics of carbon imbalance in agricultural cultivation in Hunan Province gradually diminished after 2020.

**Fig. 7:**
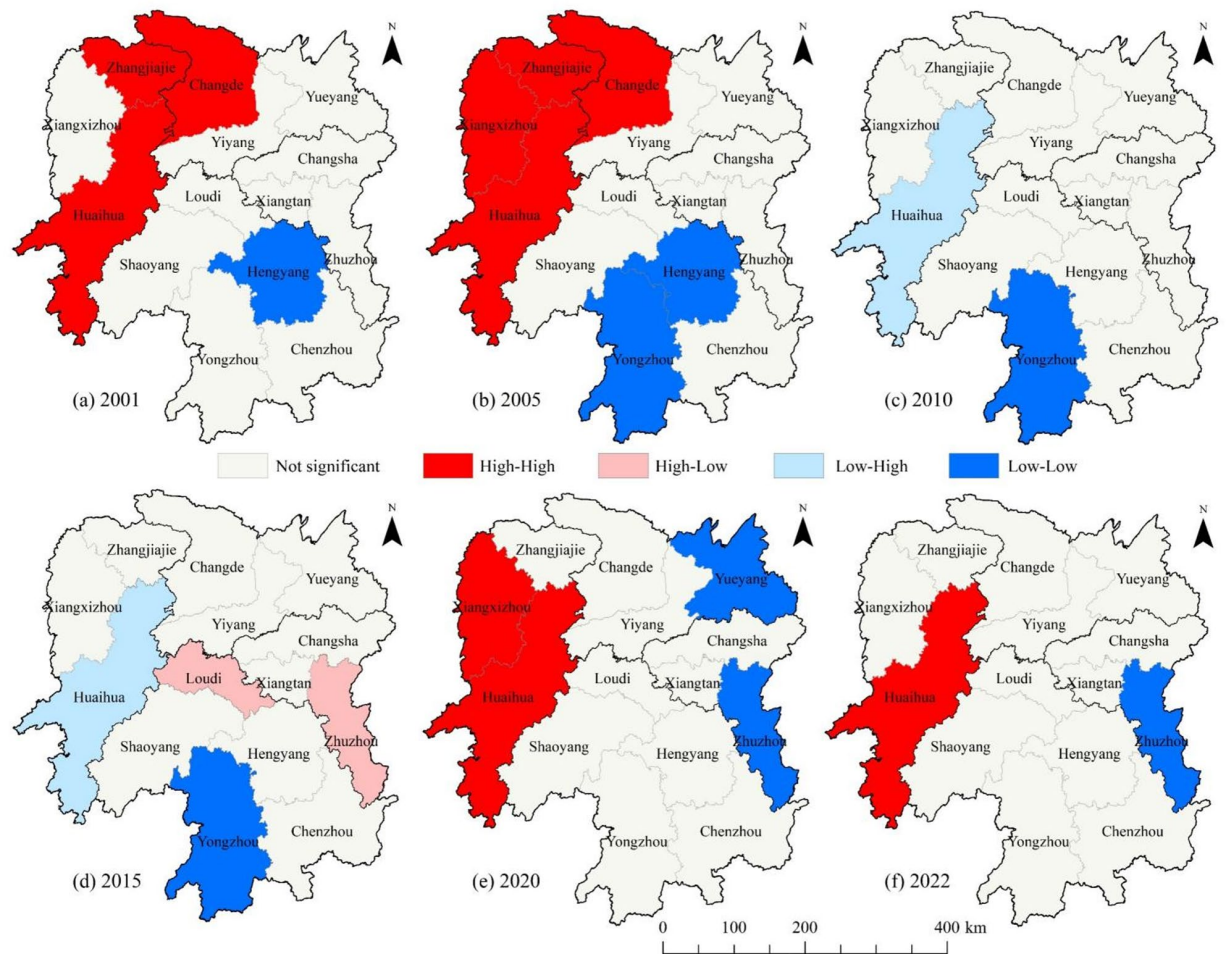
LISA cluster distribution of agricultural cultivation CII in Hunan Province

Since the global Moran’s I index cannot characterize the clustering of local spatial units, this study employs the local Moran’s I index (LISA) to reveal the changes in local spatial clustering of CII in agricultural cultivation in Hunan Province. Figure 7 illustrates the spatial clustering characteristics of agricultural cultivation CII in typical years during the study period. In 2001, the high-high clusters of agricultural cultivation CII were primarily distributed in Huaihua, Zhangjiajie, and Changde, while the low-low cluster was observed in Hengyang. In 2005, the high-high clusters expanded compared to 2001, with Xiangxizhou newly included; meanwhile, low-low clusters formed in Yongzhou and Hengyang in the Xiangnan region, and the local spatial association in the remaining eight city regions was insignificant. This indicates that the spatial clustering characteristics of agricultural cultivation CII in Hunan Province remained relatively stable from 2001 to 2005. Compared with 2005, the high-high cluster of agricultural cultivation CII disappeared in 2010, with only Yongzhou remaining as a low-low cluster, while Huaihua shifted from a high-high to a low-high cluster. This change may be attributed to the increased planting areas of wheat and corn in Changde and Huaihua in 2010, which led to higher carbon sequestration rates and a decrease in CII. This weakened the spatial correlation between Changde, Huaihua, and their surrounding city regions, resulting in the disappearance of Changde’s high-high cluster and Huaihua’s transition to a low-high cluster. In 2015, except for Loudi and Zhuzhou, which exhibited high-low clustering, the spatial pattern of CII in other city regions remained consistent with that in 2010, indicating that most city regions showed no significant spatial clustering of CII. This phenomenon may be attributed to the promotion of low-carbon concepts and technologies in Hunan Province’s agricultural sector around 2015: the reduced use of high-carbon agricultural inputs such as chemical fertilizers and pesticides led to a decrease in carbon emissions from agricultural cultivation. Additionally, variations in the implementation intensity of agricultural cultivation policies across city regions resulted in the 2015 CII spatial pattern being characterized by the coexistence of southern clustering and central dispersion. In 2020, Huaihua and Xiangxizhou exhibited high-high clustering, while Yueyang and Zhuzhou showed low-low clustering. By 2022, the high-high clustering in Xiangxizhou and the low-low clustering in Yueyang had become less pronounced, with a contraction in their clustering ranges. This indicates that the effects of Hunan Province’s green agricultural policies are gradually emerging. However, due to GPA restrictions, the Xiangxi region has formed clusters of high CII values, making emission reduction more challenging [51]. In contrast, the flat terrain in Xiangbei and Xiangdong facilitates the promotion of low-carbon technologies in large-scale agricultural cultivation, resulting in a “west-high, east-low” spatial pattern of CII in Hunan’s agricultural cultivation. Nevertheless, as policy implementation remains incompletely synchronized, a stable clustering pattern has not yet fully materialized.

**Fig. 8:**
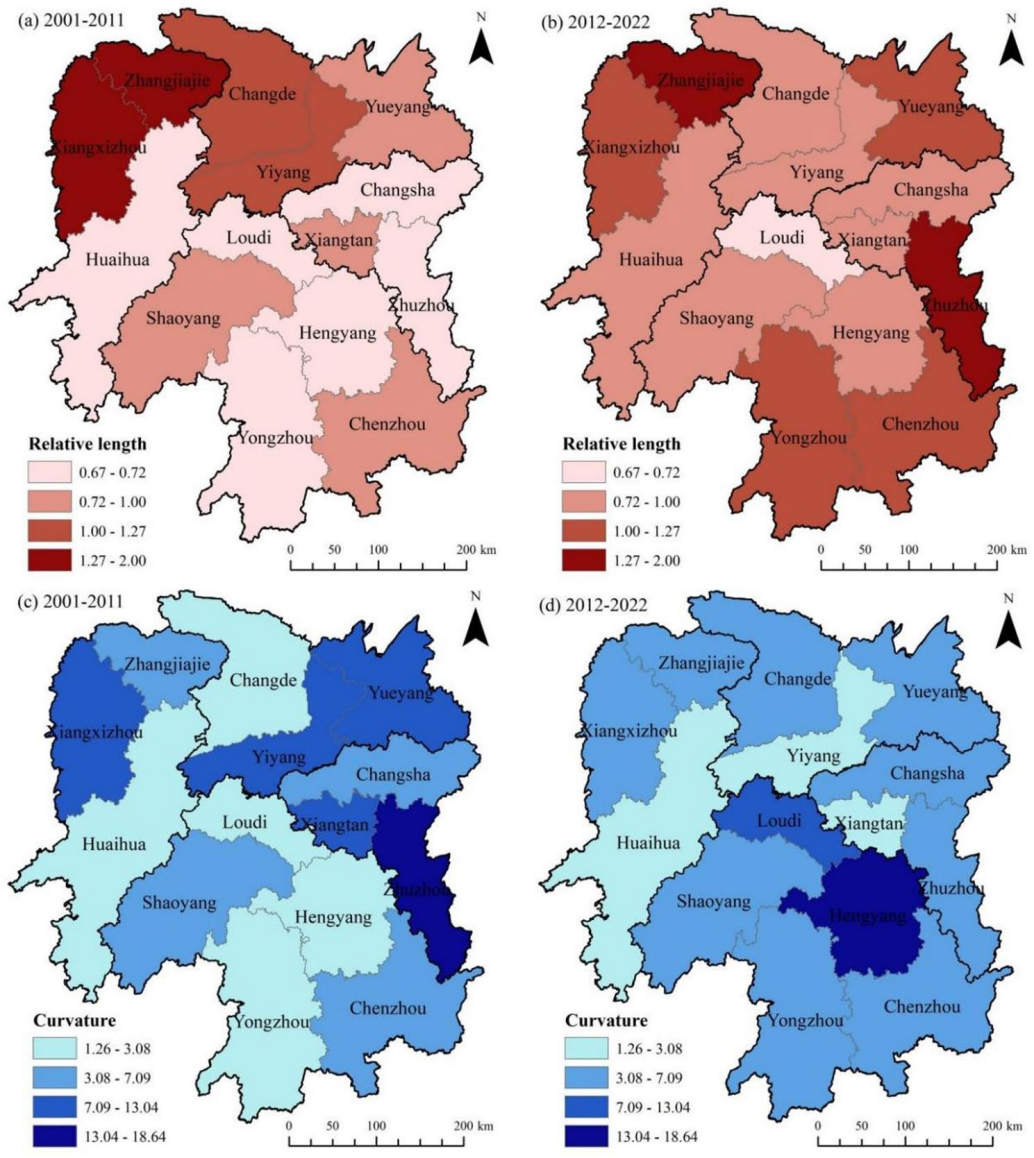
Changes in the relative length and curvature of the LISA time path of CII in agricultural cultivation across Hunan Province

To further reveal the dynamics of the local spatial structure and volatility in the direction of spatial dependence of CII in agricultural cultivation in Hunan Province, this study calculated the relative length and curvature of the LISA time path from 2001 to 2022. Using the natural breaks method in ArcGIS 10.8, combined with manual breakpoint adjustments, the results were divided into four intervals from low to high (Fig. 8). Figure 8a shows that from 2001 to 2011, 10 prefecture-level cities in Hunan Province had a relative length of the LISA time path for agricultural cultivation CII less than 1, accounting for approximately 71% of the total number of prefecture-level cities in the province. This indicates that the local spatial structure of most prefecture-level cities remained relatively stable during this period. In contrast, Xiangxizhou, Zhangjiajie, Changde, and Yiyang all had a relative length greater than 1, suggesting that the local spatial structure of these regions exhibited stronger dynamism. Compared to the previous period, between 2012 and 2022, the relative lengths of Xiangxizhou and Zhangjiajie remained above 1, while those of Yongzhou, Chenzhou, Zhuzhou, and Yueyang increased; the relative lengths of Changde and Yiyang decreased, and the number of city regions in Hunan Province with a relative length below 1 dropped to six (Fig. 8b). Additionally, the relative lengths of Huaihua and Hengyang also increased to some extent. This indicates that the dynamics of the local spatial structure of CII in most city regions of Hunan Province gradually strengthened during this period. This may be attributed to Hunan Province’s active response, since 2012, to national initiatives promoting eco-friendly agriculture and technological advancements in the agricultural sector [50], which led to reduced use of agricultural inputs. This lowered carbon emission rates in most city regions, thereby affecting the stability of the local spatial structure of agricultural cultivation CII in each region. Figure 8c shows that Zhuzhou exhibited the highest curvature of the LISA time path from 2001 to 2011, indicating that its CII changes displayed strong volatility and were significantly influenced by the spatiotemporal dependence of local structures. Next, Xiangxizhou, Yiyang, Yueyang, and Xiangtan had relatively high curvature, suggesting high volatility in both their CII changes and local spatial dependence. In contrast, Zhangjiajie, Changsha, Shaoyang, and Chenzhou showed low curvature, with relatively weak volatility in CII changes, while the remaining city regions exhibited moderate curvature. From 2012 to 2022, except that Hengyang became the city region with the highest curvature in Hunan Province, the number of city regions with higher or lower curvature both decreased, while those with medium curvature increased from 4 to 9 (Fig. 8d). Such a trend implies that during this period, there was a reduction in city regions with either weak or strong local spatiotemporal dependence, with a shift toward medium curvature. This indicates that changes in the spatial dependence of CII in agricultural cultivation across most city regions of Hunan Province remained relatively stable.

To reflect the mutual transitions of local spatial association types in the Moran scatter plot, this paper employs spatiotemporal transitions to classify the CII transition states between each city region and its adjacent ones into 4 types [38]: Type Ⅰ indicates that both the city region itself and its neighboring regions remain stable, including HH_t_→HH_t+1_、HL_t_→HL_t+1_、LL_t_→LL_t+1_ and LH_t_→LH_t+1_; Type Ⅱ indicates that the city region itself undergoes a transition, including HH_t_→LH_t+1_, HL_t_→LL_t+1_, LH_t_→HH_t+1_, and LL_t_→HL_t+1_; Type Ⅲ indicates that the adjacent units of the city region undergo transitions, including HH_t_→HL_t+1_, HL_t_→HH_t+1_, LH_t_→LL_t+1_, and LL_t_→LH_t+1_; Type Ⅳ indicates that both the city region itself and its adjacent regions undergo transitions, including HH_t_→LL_t+1_, HL_t_→LH_t+1_, LL_t_→HH_t+1_, and LH_t_→HL_t+1_. Table 5 shows that Type I transitions occurred most frequently, with 38 instances, accounting for approximately 69% of all transitions. This indicates that the interannual spatiotemporal transitions of CII in agricultural cultivation across most city regions in Hunan Province were relatively stable. Type II transitions occurred 7 times, with 6 of these in Huaihua, indicating significant instability in Huaihua’s agricultural cultivation CII, potentially due to frequent changes in its grain cultivation patterns; other transition types did not occur. Overall, the evolution of the spatial correlation structure of agricultural cultivation CII in Hunan Province is relatively slow, and the spatiotemporal transitions of CII across city regions exhibit certain spatial lock-in characteristics.

**Table 5:** Spatiotemporal transitions of local moran’s I for CII across City regions in Hunan Province

	HH_t+1_	LH_t+1_	HL_t+1_	LL_t+1_
HH_t_	I (16)	II (3)	III (0)	IV (0)
LHt	II (3)	I (10)	IV (0)	III (0)
HL_t_	III (0)	IV (0)	I (8)	II (1)
LL_t_	IV (0)	III (0)	II (0)	I (14)

The numbers in parentheses indicate the number of Local Moran’s I transitions for each city during the study period; t denotes the year; HH stands for high–high, LH for low–high, HL for high–low, and LL for low–low

### **Analysis of driving factors for agricultural cultivation CII**

#### Identification of the importance of driving factors

Table 6 presents the PLS-VIP results for the agricultural cultivation CII in Hunan Province. The analysis identifies GPA, GST, IARDF, PAOV, FUI, and PUI as key drivers of the CII, with their VIP values all exceeding 1. Among them, the regression coefficients for GPA, GST, and IARDF are − 0.333, −0.151, and − 0.117, respectively, indicating that they have an inhibitory effect on the agricultural cultivation CII. This implies that large-scale cultivation, favorable climatic conditions, and reasonable agricultural R&D investment can effectively enhance the carbon sequestration capacity of crops and soil during agricultural cultivation, narrow the gap between carbon sequestration and emissions, and thereby mitigate carbon imbalance. In contrast, the regression coefficients for PAOV, FUI, and PUI were 0.195, 0.179, and 0.162, respectively, indicating they have a positive driving effect on agricultural cultivation CII and promote its growth. This suggests that increased use of agricultural inputs (e.g., fertilizers and pesticides) elevates carbon emissions from agricultural planting, exacerbating the risk of carbon imbalance. Furthermore, the VIP values of IAF, PAMP, GDR, FSA, and GSP were all less than 1, with their regression coefficients exhibiting small absolute values, indicating they are not significant factors influencing agricultural cultivation CII.

**Table 6:** Regression coefficients and VIP values of various driving factors

Factors	Coefficient	VIP	Factors	Coefficient	VIP	Factors	Coefficient	VIP
GPA	−0.333	1.446	FUI	0.179	1.149	GDR	0.030	0.522
GST	−0.151	1.412	PUI	0.162	1.044	FSA	−0.034	0.504
IARDF	−0.117	1.195	IAF	0.072	0.776	GSP	−0.087	0.283
PAOV	0.195	1.150	PAMP	0.018	0.740			

#### Spatiotemporal heterogeneity of driving factors

**Fig. 9:**
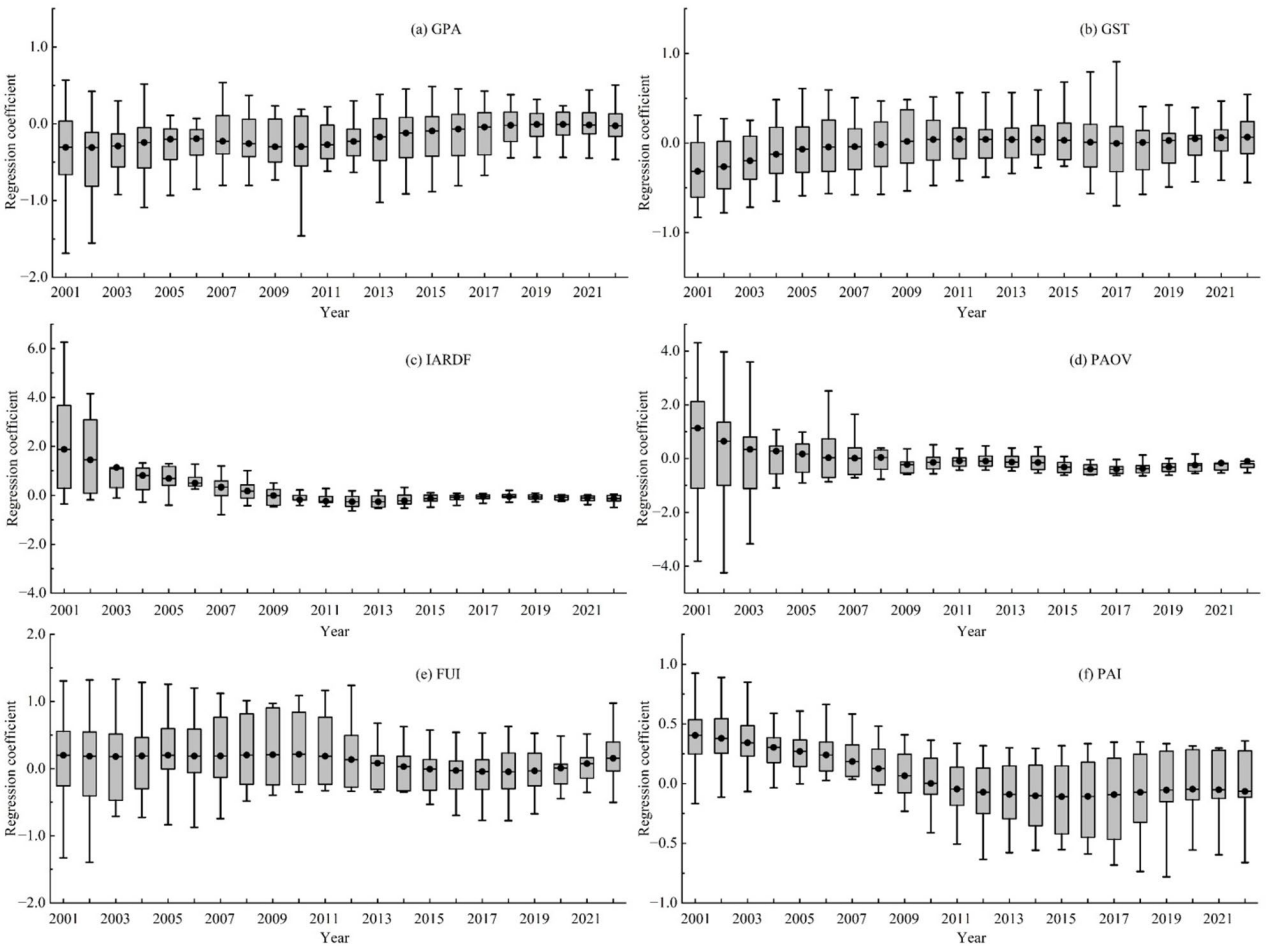
Time variation of regression coefficients for CII driving factors (black solid dots in the figure represent the average values of regression coefficients for each city in the same year)

**Fig. 10:**
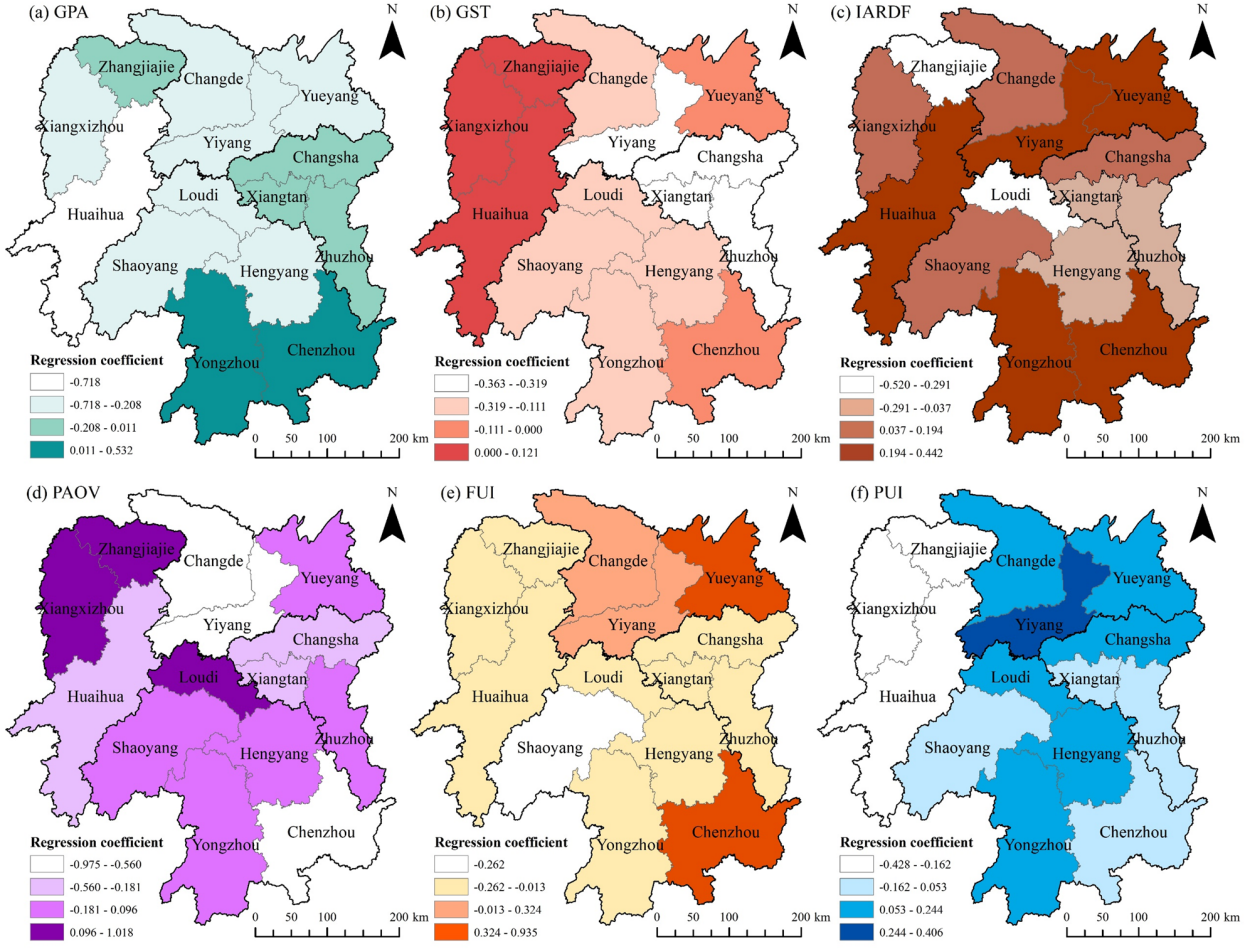
Spatial pattern of regression coefficients for CII drivers in agricultural cultivation (the regression coefficients for each city in the figure are the average values for the study period)

To examine the spatiotemporal heterogeneity in the drivers of agricultural cultivation CII across Hunan Province, this study selects six driving factors (GPA, GST, IARDF, PAOV, FUI, and PUI) with VIP values greater than 1 as independent variables, with agricultural cultivation CII for each city as the dependent variable, and employs the GTWR model for regression analysis. Furthermore, to prevent multicollinearity among driving factors from distorting regression results, a collinearity test was performed on the selected independent variables prior to model implementation. All independent variables had VIF values < 10, indicating the absence of multicollinearity and their suitability for model analysis. Meanwhile, a comparison of the GTWR and GWR models revealed that the GTWR model (R² = 0.92, AICc = 398.47) significantly outperformed the GWR model (R² = 0.76, AICc = 477.80), confirming its greater suitability for analyzing spatiotemporal heterogeneity. Figures 9 and 10 illustrate significant spatiotemporal heterogeneity in the influence of each factor on agricultural cultivation CII, with detailed analyses provided below.

Figure 9a shows that the mean regression coefficients of GPA across cities in Hunan Province exhibited a negative effect from 2001 to 2022, ranging from − 0.309 to −0.008. This may be attributed to increased carbon uptake by food crops as GPA expands, which partially inhibits the growth of agricultural cultivation CII. Furthermore, the implementation of low-carbon agricultural policies has likely encouraged governments and farmers to invest more in low-carbon agricultural technologies, reducing carbon emissions during cultivation through measures such as input reduction and efficiency improvement—thereby further suppressing CII growth. In conjunction with Fig. 10a, GPA exerts an inhibitory effect on agricultural cultivation CII in most Hunan cities, consistent with the temporal variation in GPA regression coefficients. However, in cities including Changsha, Yongzhou, and Chenzhou, the mean regression coefficient of GPA remains positive. This may stem from their early high reliance on agricultural mechanization. As GPA expands, fuel consumption and total agricultural machinery power increase, resulting in a greater rise in carbon emissions than in carbon uptake, thereby driving CII growth. Notably, with the advancement of green agriculture, the positive influence of GPA on agricultural cultivation CII in these cities is gradually transitioning to a negative effect.

Figure 9b shows that the average change in the GST regression coefficient during the study period ranged from − 0.316 to 0.044. Due to temperature directly or indirectly affecting crop growth by influencing soil moisture and organic matter content, both excessively high and low temperatures are detrimental to carbon absorption by crops and soil carbon sequestration during agricultural cultivation [7]. Therefore, GST had a predominantly negative impact in most years. As a major rice-growing province in China, Hunan Province exhibited GST variations of 24.1–26.8 °C across its cities during the study period, which generally aligns with the optimal temperature range for rice growth (25.0–28.0 °C) [52]. It provides suitable conditions for rice growth and enhanced carbon sink capacity across Hunan’s cities, thereby partially suppressing the growth of agricultural cultivation CII. Spatially, GST exerts a positive effect in Xiangxizhou, Huaihua, and Zhangjiajie, but a negative effect in Changsha, Zhuzhou, Xiangtan, and Yiyang (Fig. 10b). This pattern arises because the Xiangxi region, with relatively high elevation, has an average temperature of 22.1–22.6 °C during the rice-growing season—inhibitory to rice growth—reducing crop and soil carbon uptake capacity and promoting CII. Conversely, Changsha, Zhuzhou, Xiangtan, and Yiyang, characterized by flat terrain and favorable agricultural conditions, enhance crop and soil carbon sink capacity, thereby significantly suppressing agricultural cultivation CII.

The effect of IARDF on agricultural cultivation CII was positive between 2001 and 2008, with mean regression coefficients ranging from 0.176 to 1.871. However, between 2009 and 2022, these coefficients shifted to negative values, ranging from − 0.259 to −0.006 (Fig. 9c). This temporal shift may be attributed to the earlier policy context, where IARDF primarily focused on boosting grain production and farmers’ income, fostering a high-input, high-output agricultural model that increased agricultural carbon emissions and thus drove up CII. In contrast, with the advancement of green agriculture in the later period, R&D investments shifted toward low-carbon technological innovations (e.g., precision irrigation, fertilizer reduction with efficiency gains, high-efficiency agricultural machinery, and crop variety improvement), significantly reducing agricultural carbon emissions and thereby inhibiting CII. Spatially, IARDF exerts a significant negative effect across most municipal areas in Hunan Province, while its influence is relatively weaker in Huaihua, Yiyang, Yueyang, Yongzhou, and Chenzhou (Fig. 10c). This phenomenon is primarily attributed to large-scale grain cultivation in cities like Huaihua and Yiyang, where the inertia of traditional farming practices hinders the transition to green agricultural technologies. It makes the adaptation and promotion of low-carbon technologies require a longer timeline, delaying the inhibitory effect of IARDF on agricultural cultivation CII, which gradually became evident after 2016.

The mean regression coefficient of PAOV ranged from 0.013 to 1.128 between 2001 and 2008, but after 2009, it narrowed to near zero and began exerting a weak negative effect on agricultural cultivation CII. Specifically, the mean regression coefficient for 2009–2022 ranged from − 0.227 to −0.094 (Fig. 9d). This suggests that early increases in PAOV were likely accompanied by high-carbon agricultural practices, such as overreliance on agricultural inputs and mechanized farming, which promoted the growth of agricultural cultivation CII. However, the post-2009 transformation of agricultural development models—via quality improvement, efficiency enhancement, and green production technologies—gradually decoupled PAOV growth from agricultural cultivation CII, significantly weakening its positive driving effect. As shown in Fig. 10d, PAOV exerts a strong positive effect in Zhangjiajie, Xiangxizhou, and Loudi, but a negative effect in Changde, Yiyang, and Chenzhou. This pattern is primarily attributed to the relatively poor green agricultural transformation in Zhangjiajie, Xiangxizhou, and Loudi, where PAOV improvements remain linked to high-input production practices—exacerbating agricultural cultivation CII. In contrast, agricultural activities in Changde, Yiyang, and Chenzhou have largely achieved scale; through smart agriculture development and crop structure adjustments, these regions have enhanced agricultural modernization, enabling PAOV growth to align with low-carbon development and gradually suppress CII growth.

Between 2001 and 2022, the mean regression coefficient of FUI ranged from − 0.045 to 0.214, with its overall driving effect on agricultural cultivation CII exhibiting slight fluctuations (Fig. 9e). This pattern primarily stems from the absence of explicit national fertilizer reduction targets prior to 2015. Consequently, fertilizer usage in Hunan Province increased at an annual rate of 1% from 2001 to 2015, and carbon emissions from fertilizer application contributed to rising CII. However, following the 2015 introduction of the Implementation Plan for Zero Growth in Fertilizer Use in Hunan Province by 2020, the province promoted fertilizer reduction and efficiency through measures such as soil-testing-based formulated fertilization and organic fertilizer substitution pilots. These efforts achieved a decline in fertilizer usage, gradually weakening FUI’s positive effect on CII. Additionally, the 2020 global food crisis warning heightened domestic food security concerns, prompting a 2.1% expansion in Hunan’s grain sown area (relative to the previous year) and a 1.5% year-on-year increase in fertilizer application—thereby driving a renewed rise in FUI’s regression coefficient. Spatially (Fig. 10e), FUI exerted the strongest positive effect in Yueyang and Chenzhou, followed by Changde and Yiyang. This is likely because regions with higher grain yields have greater fertilizer demand, such that increased FUI is more prone to elevating CII in these areas.

The mean regression coefficient of PUI during the study period ranged from − 0.109 to 0.405, exhibiting a more pronounced downward trend compared to FUI (Fig. 9f). This stems primarily from earlier implementation of pesticide control policies in Hunan Province. For instance, the Maximum Residue Limits for Pesticides in Agricultural Products (2003) and Measures for the Administration of Agricultural Product Quality and Safety (2005) explicitly banned highly toxic pesticides and established a pesticide residue monitoring system. The 2015 introduction of the “Dual Reduction of Pesticides and Fertilizers” policy further drove down PUI. Between 2001 and 2022, pesticide usage in Hunan Province decreased from 1.2 × 10⁵ tons to 7.47 × 10⁴ tons, with a 39.3% reduction—far exceeding the 13.4% decline in fertilizer usage over the same period—resulting in a marked downward trend in PUI’s regression coefficient. As shown in Fig. 10f, PUI exerted a significant positive effect on CII in Yiyang, Changde, Yueyang, Changsha, Loudi, Hengyang, and Yongzhou, whereas coefficients in the Xiangxi region were generally lower. This spatial pattern likely arises because areas like Yiyang have a larger share of rice cultivation in Hunan; rice is prone to pests and diseases, increasing pesticide demand, thus strengthening the correlation between PUI and agricultural cultivation CII. In contrast, the smaller rice cultivation area in the Xiangxi region inherently reduces pesticide usage, weakening PUI’s driving effect.

## Discuss

### Discussion on the Spatiotemporal evolution of CII in agricultural cultivation

An analysis of the temporal evolution of the agricultural cultivation CII in Hunan Province from 2001 to 2022 reveals that the carbon budget of agricultural cultivation in the province has long been in a state of carbon imbalance, although the CII has exhibited a fluctuating downward trend. As a typical main rice-producing region in China, Hunan Province is similar to subtropical rice-producing regions such as the Ganges Plain in India and the Mekong Delta in Thailand, and the large amount of CH₄ emissions generated during rice cultivation (about 58.75% of the total carbon emissions from agricultural cultivation [49]) greatly exacerbates the risk of carbon imbalance from agricultural cultivation [53, 54]. Driven particularly by the goals of increasing grain production and raising farmers’ incomes, the agricultural cultivation CII in Hunan Province peaked in 2005. After 2015, however, the province actively promoted sustainable agriculture and vigorously implemented initiatives to reduce chemical fertilizer and pesticide application while improving their efficiency. This led to a significant reduction in agricultural input use, which in turn slowed the growth rate of carbon emissions from agricultural cultivation. Consequently, the CII began to decline in 2017—a trend that aligns closely with the simultaneous downward trend of agricultural carbon emissions across China [55].

From a spatial perspective, although the Xiangbei and Xiangdong regions are major grain-producing regions in Hunan Province, the agricultural cultivation CII in these areas has been effectively curbed in recent years. This is attributed to the strong emphasis on green and low-carbon agriculture, coupled with the adoption of emission reduction measures such as water-saving irrigation and soil testing-based formula fertilization. Together with relevant research findings from regions like the Yangtze River Delta in China, this practical conclusion confirms that cultivation models centered on the application of low-carbon technologies are an effective pathway to mitigate agricultural carbon imbalance [56]. In the Xiangnan region, most cities’ CII values exhibit an M-shaped variation. Specifically, in Loudi and Yongzhou, the increase in agricultural film and diesel fuel usage has driven an upward trend in the CII of agricultural cultivation. This further verifies the positive driving effect of agricultural input intensity on CII, while also highlighting the potential threat of traditional high-carbon agricultural production models to carbon balance. In the Xiangxi region (particularly prominent in Xiangxizhou and Zhangjiajie), frequent changes in GPA have weakened soil carbon sequestration capacity, resulting in long-term severe carbon imbalance in the CII of each municipality. This phenomenon is analogous to the carbon imbalance lock-in effect caused by topographic constraints in mountainous regions of Nepal and the Andean Mountains of Peru [57, 58].

Based on the spatial variation trends, the early spatial correlation structure of the agricultural cultivation CII in Hunan Province evolved slowly, with a pronounced spatial solidification effect. However, driven by policy interventions, the clustering pattern of agricultural cultivation CII shifted from “high in the north and low in the south” to “high in the west and low in the east,” and the volatility of CII changes gradually intensified. This spatial pattern is mainly attributed to recent years’ adjustments in agricultural cultivation structures and the promotion of low-carbon technologies (e.g., precision fertilization, pesticide reduction, and efficiency enhancement) in the Xiangbei and Xiangdong regions. These measures have facilitated the transition of CII from moderate to mild carbon imbalance, thereby forming clustered areas of mild carbon imbalance. This also indirectly demonstrates the effectiveness of policy interventions in curbing agricultural cultivation CII [59, 60]. In the Xiangxi region, the fragmentation of arable land coupled with irrational agricultural cultivation practices has locked the region into a high-value CII cluster. Additionally, the absence of a corresponding ecological compensation mechanism has resulted in the long-term failure to effectively address the carbon imbalance in local agricultural cultivation. This indicates that transforming the carbon imbalance in the Xiangxi region agricultural cultivation is particularly challenging, though the region also holds significant emission reduction potential [25]. In the Xiangnan region, except for Loudi, the agricultural cultivation CII of other cities remains in a mild and relatively stable state of carbon imbalance. For Loudi, during 2015–2020, urbanization and industrialization encroached on arable land, leading to a reduction in GPA and a subsequent weakening of the carbon sequestration capacity of agricultural cultivation—ultimately causing a resurgence in CII. This pattern closely mirrors the carbon imbalance observed in Southeast Asia and China’s Pearl River Delta, where rapid urbanization and industrialization have similarly squeezed arable land, and collectively reflects the intensifying role of the process of non-agriculturalization on the carbon imbalance of agriculture [61, 62].

### Policy implications of Spatiotemporal heterogeneity in drivers

Identifying the driving factors of the agricultural cultivation CII is a prerequisite for addressing the agricultural carbon imbalance. Analyses of the PLS-VIP method and GTWR model results indicate that the GPA exerts the strongest negative impact—specifically, the expansion of GPA during agricultural cultivation helps increase the carbon sequestration capacity of crops and soil, thereby curbing the growth of CII. This finding provides robust theoretical support for the theory that agricultural cultivation promotes carbon sequestration and emission reduction. In contrast, the PAOV has the most significant positive effect on the agricultural cultivation CII, reflecting that the growth of PAOV in Hunan Province is typically accompanied by high carbon emissions from excessive agricultural inputs, which exacerbates the agricultural cultivation CII. Therefore, how to achieve coordinated development between agricultural economic growth and the mitigation of carbon imbalance has become a critical issue that urgently needs to be addressed for sustainable agricultural development. GST exerts an inhibitory effect on CII in most cities of Hunan Province, which deviates from the findings of Komiya et al. regarding carbon emissions from rice cultivation in Thailand [63]. As a tropical monsoon region, Thailand experiences high temperatures and humidity during the rice-growing season—conditions that tend to intensify CH_4_ emissions from paddy fields, thereby inducing a driving effect of GST on carbon imbalance. In contrast, Hunan Province, located in the subtropical monsoon zone, exhibits temperature variations within the optimal growth range for rice and other grain crops. Moderate temperature increases in this context can enhance crop photosynthesis, thereby strengthening farmland carbon sequestration capacity and consequently restraining CII growth. It is worth noting that whether the driving direction of GST in Hunan Province will shift with the further intensification of global warming remains to be further verified. Additionally, IARDF, FUI, and PUI also exert significant influences on CII, which aligns with the conclusions of most studies on drivers of agricultural carbon emissions, though the magnitude of these effects varies across regions [9, 31].

From a temporal perspective, 2009 marked a critical transition point for agricultural cultivation in Hunan Province. The negative effect of IARDF began to emerge, while the positive driving roles of the PAOV, FUI, and PUI weakened. This observation underscores the effectiveness of a series of low-carbon agricultural transition policies in China, such as agricultural low-carbon pilot programs and the dual reduction of pesticide and fertilizer use. Therefore, moving forward, the regulatory role of IARDF should be further strengthened, with targeted support for the research and development of low-carbon technologies (e.g., precision irrigation and biopesticides) to continuously enhance its negative inhibitory effect on agricultural carbon imbalance. In particular, for Loudi—a city highly dependent on agricultural machinery fuel consumption—appropriate measures (e.g., agricultural machinery upgrades and low-carbon subsidies) can be implemented to reduce the carbon emission intensity of agricultural equipment. Meanwhile, the shift in PAOV’s driving effect during this period is also consistent with the broader trend of China’s agricultural economic development gradually decoupling from carbon imbalance [60, 64]– [65]. In response to the transient rebound in the FUI driving coefficient in 2020, it is imperative to establish a synergistic mechanism between food security and low-carbon agricultural development to prevent the resurgence of the driving effect of high-carbon agricultural inputs.

Furthermore, based on the spatial differentiation characteristics of the CII driving effect, differentiated agricultural carbon sequestration and emission reduction strategies can be formulated for Hunan Province. For instance, in the Xiangbei region, the focus should be on regulating FUI and PUI and promoting soil testing-based formula fertilization and biopesticide research and development, while intensifying efforts in biopesticide application to reduce carbon emissions from high-carbon agricultural inputs at the source; In the Xiangxi region, targeted measures are needed to address fragmented arable land and weak carbon sequestration capacity, such as enhancing regional carbon sequestration capacity through farmland consolidation and ecological compensation mechanisms; In the Xiangdong region, leveraging its advantage in high R&D investment, the region should accelerate the deployment and promotion of more mature low-carbon technologies, with technological iteration driving the mitigation of carbon imbalance; In the Xiangnan region, differentiated policies are essential. Particularly in Loudi, efforts should prioritize adjusting the agricultural cultivation structure and retrofitting agricultural machinery for low-carbon operation, to specifically tackle the rebound of carbon imbalance caused by urbanization encroaching on arable land and traditional cultivation patterns.

### Limitations

Compared with existing research, this study, to some extent, moves beyond the traditional static framework that evaluates carbon imbalance based on the difference or ratio between carbon emissions and carbon sequestration. Specifically, it constructs a dynamic index to characterize the carbon imbalance in agricultural cultivation and analyzes its spatiotemporal variations and the heterogeneity of driving factors. Nevertheless, this study still has several limitations. First, in the estimation of carbon emissions and carbon sequestration from agricultural cultivation, some correlation coefficients were derived from existing literature rather than direct measurements, which may introduce uncertainties into the research results. Future research could incorporate field observations or remote-sensing data to verify these findings. Second, due to data availability constraints, the analysis of drivers for agricultural carbon imbalance primarily focused on factors such as production inputs and land use, while excluding difficult-to-quantify elements like policy implementation efficiency and agricultural technology dissemination from the analytical framework. Future studies could conduct more systematic analyses by integrating agricultural policy and technological factors.

## Conclusions

Based on the calculation of the CII for agricultural cultivation across cities in Hunan Province from 2001 to 2022, this study investigates the spatiotemporal variation patterns and the spatiotemporal heterogeneity of the driving factors. The main conclusions are as follows:

(1) Over time, agricultural cultivation in Hunan Province has long been in a state of carbon imbalance, with CII showing an overall downward trend but with periodic fluctuations. Regionally, the CII fluctuation range in the Xiangbei region and the Xiangdong region gradually narrowed, exhibiting a downward trend. In the Xiangnan region, Loudi and Yongzhou were exceptions, with their CII showing a slow upward trend; CII in other cities fluctuated slightly, with no significant overall trend. The Xiangxi region experienced significant CII fluctuations due to frequent conversions between tourism land and agricultural land.

(2) Spatially, agricultural cultivation CII in Hunan Province generally transitioned from a “north-high, south-low” pattern to a “west-high, east-low” pattern. The imbalance center shifted from Yiyang to Loudi, with a continuous southwestward movement. From 2001 to 2022, areas with mild carbon imbalance gradually expanded, while regions with severe and extreme carbon imbalance steadily contracted. During the study period, the spatial correlation of CII in Hunan Province evolved through three stages: significant positive correlation, random distribution, and weak positive correlation. Furthermore, the spatial distribution pattern of local LISA aggregates also shows a shift from “high in the north and low in the south” to “high in the west and low in the east.”

(3) In terms of LISA time paths and spatiotemporal transitions, Xiangxizhou and Zhangjiajie exhibited the strongest CII fluctuations, whereas cities in eastern Hunan showed increasing CII stability. Moreover, Type I transitions accounted for 69% of total transitions, indicating strong stability in the spatiotemporal patterns of agricultural cultivation CII across Hunan’s cities, with slow changes in their spatial correlation structures.

(4) In terms of driving factors, the key factors influencing agricultural cultivation CII in Hunan Province are GPA, GST, IARDF, PAOV, FUI, and PUI. GPA, GST, and IARDF effectively inhibit agricultural cultivation CII, with IARDF’s negative effect becoming pronounced post-2009. PAOV, FUI, and PUI initially promoted agricultural cultivation CII, but their driving effects weakened after 2011. Spatially, FUI and PUI exert a greater influence in the Xiangbei region, while the Xiangdong region is significantly affected by GPA and GST. GPA strongly drives agricultural cultivation CII in the Xiangnan region (excluding Loudi), whereas IARDF and PAOV more prominently influence Loudi. The Xiangxi region is primarily affected by GST and IARDF.

## Data Availability

Generated datasets generated and analyses are available from the corresponding authors on reasonable request.
